# ZNF322A-mediated protein phosphorylation induces autophagosome formation through modulation of IRS1-AKT glucose uptake and HSP-elicited UPR in lung cancer

**DOI:** 10.1186/s12929-020-00668-5

**Published:** 2020-06-23

**Authors:** Chantal Hoi Yin Cheung, Chia-Lang Hsu, Tsai-Yu Lin, Wei-Ting Chen, Yi-Ching Wang, Hsuan-Cheng Huang, Hsueh-Fen Juan

**Affiliations:** 1 Department of Life Science, National Taiwan University, Taipei, 10617 Taiwan; 2 Department of Medical Research, National Taiwan University Hospital, Taipei, 10002 Taiwan; 3 Department of Pharmacology, College of Medicine, National Cheng Kung University, Tainan, 70101 Taiwan; 4 Institute of Basic Medical Sciences, College of Medicine, National Cheng Kung University, Tainan, 70101 Taiwan; 5 Institute of Biomedical Informatics, National Yang-Ming University, No.155, Sec.2, Linong Street, Taipei, 11221 Taiwan; 6 Department of Life Science, Graduate Institute of Biomedical Electronics and Bioinformatics, National Taiwan University, No. 1, Sec. 4, Roosevelt Road, Taipei, 10617 Taiwan

**Keywords:** Autophagy, Glucose starvation, Heat stress, Lung cancer, Phosphoproteomics, Proteomics, Zinc-finger protein

## Abstract

**Background:**

ZNF322A is an oncogenic transcription factor that belongs to the Cys2His2-type zinc-finger protein family. Accumulating evidence suggests that ZNF322A may contribute to the tumorigenesis of lung cancer, however, the ZNF322A-mediated downstream signaling pathways remain unknown.

**Methods:**

To uncover ZNF322A-mediated functional network, we applied phosphopeptide enrichment and isobaric labeling strategies with mass spectrometry-based proteomics using A549 lung cancer cells, and analyzed the differentially expressed proteins of phosphoproteomic and proteomic profiles to determine ZNF322A-modulated pathways.

**Results:**

ZNF322A highlighted a previously unidentified insulin signaling, heat stress, and signal attenuation at the post-translational level. Consistently, protein-phosphoprotein-kinase interaction network analysis revealed phosphorylation of IRS1 and HSP27 were altered upon ZNF322A-silenced lung cancer cells. Thus, we further investigated the molecular regulation of ZNF322A, and found the inhibitory transcriptional regulation of ZNF322A on PIM3, which was able to phosphorylate IRS1 at serine^1101^ in order to manipulate glucose uptake via the PI3K/AKT/mTOR signaling pathway. Moreover, ZNF322A also affects the unfolded protein response by phosphorylation of HSP27^S82^ and eIF2a^S51^, and triggers autophagosome formation in lung cancer cells.

**Conclusions:**

These findings not only give new information about the molecular regulation of the cellular proteins through ZNF322A at the post-translational level, but also provides a resource for the study of lung cancer therapy.

## Background

Cys2His2 (C2H2)-type zinc finger (ZNF) is one of the most common DNA-binding motifs and plays a major role as transcription factor, which is encoded by 2% of human genes [1]. Cumulative evidence shows that the ZNFs fulfill a variety of regulatory roles in cellular processes, including differentiation, development, metabolism, cell survival, apoptosis, and autophagy [2–6]. Besides, C2H2-ZNFs have been reported as either activator or suppressor, depending on the cellular context and target genes. These findings suggest that C2H2-ZNFs are pivotal regulators in tumorigenesis. In our previous study, ZNF322A, a protein belongs to C2H2-ZNF family, contributes to cell proliferation, motility, tumor growth, metabolism, stemness properties, and metastasis [7–9]. Mechanistically, ZNF322A activates the transcriptional activity of serum response element and activator protein-1, and cooperates with c-Jun to activate alpha-adducin and cyclin D1 [7]. Clinically, ZNF322A was found significantly amplified in Asian and Caucasian lung cancer patients with poor prognosis [10]. Our previous quantitative proteomic analysis of ZNF322A-silenced lung cancer cells revealed that the downstream were participated in signal transduction and protein phosphorylation, vesicle-mediated transport, generation of energy and chromatin organization [7]. These findings indicate that ZNF322A plays an important role in lung cancer development, however, the regulatory role of ZNF322A at the phosphorylation level is still unknown.

Cancer is a set of diseases characterized by abnormal alterations that leads to unrestricted cell growth, enhanced metabolism, and suppression of programmed cell death. Dysregulation of kinases and phosphatases is commonly associated with various cancers, thereby conferring the cancer cells with proliferation advantages [11, 12]. Protein phosphorylation is one of the most widespread modes of post-transcriptional modification (PTM) in cell signaling. Many protein kinases such as mitogen-activated protein kinases (MAPKs) and the serine/threonine protein kinase Akt (AKT) are major enzymes that relay signals in response to oncogenic stimuli and drive transcriptional programs in favor of tumor growth [13, 14]. Therefore, characterization of protein phosphorylation induced signaling changes may provide insights into the regulation of molecular mechanisms involved in tumor progression. Quantitative phosphoproteomic profiling allows researchers to study the aberrant regulation of signaling pathways and also assists in the discovery of appropriate therapeutic targets for diseases [15–17]. With the development of phosphopeptide enrichment strategy and quantitative mass spectrometry-based proteomic analysis, it is possible to profile site-specific phosphorylation events and investigate aberrantly activated signaling pathways in cancer.

Evidence has shown that oncogenic activation of mammalian target of rapamycin (mTOR) signaling induces cellular processes required for cancer cell growth and survival [18]. Among them, the phosphatidylinositol 3-kinase/protein kinase B/mammalian target of rapamycin (PI3K/AKT/mTOR) and MAPK signaling pathways are crucial for autophagosome formation in cancer cells. PI3K/AKT signaling plays critical roles in cellular physiology and processes, such as glucose homeostasis, lipid metabolism, and cell proliferation. Interestingly, knockdown of AKT reduce insulin-induced glucose uptake by regulating glucose transport through the PI3K/AKT signaling pathway [19]. Autophagy is triggered during nutrient deprivation in cells as a homeostatic response to stress [20]. During autophagosome formation, phagosome converts to autophagosome by autophagic stimulus and stress, and its maturation is completed upon fusion with lysosome [21–24]. Endoplasmic reticulum (ER) is a dynamic organelle that responses to environmental stress through a series of signaling cascades known as the unfolded protein response (UPR). Alteration of ER homeostasis causes accumulation of misfolded or unfolded proteins in the ER, which leads to the ER stress and UPR activation. Recent researches have demonstrated that the activation of the PI3K/AKT signaling pathway occurs in 90% of non-small cell lung cancer (NSCLC) cell lines, and the inhibition of this signaling cascade is not only important for induction of autophagic cell death but also provides insight for developing new treatments [25, 26].

## Methods

### Cell culture

Human lung carcinoma A549 cells (CCL-185) and H1299 cells (CRL-5803) were obtained from the American Type Culture Collection (Manassas, VA, USA). Cells were maintained in Dulbecco’s Modified Eagle’s Medium (DMEM, Gibco Laboratories, Grand Island, NY, USA) with 10% fetal bovine serum (Biological Industries, Kibbutz Beit Haemek, Israel) at 37 °C in a humidified incubator with 5% CO_2_.

### RNA interference

A549 cells and H1299 cells were transfected for 48 h with control (siControl) or ZNF322A (siZNF322A) siRNA by Lipofectamine 3000 (Invitrogen, Waltham, MA, USA) according to the manufacturer’s protocol. Transfected cells were harvested at 48 h post-transfection for further assays (Additional file 1: Fig. S1).

### RNA extraction and cDNA synthesis

Total RNA of A549 cells and H1299 cells was extracted by Direct-zol™ RNA MiniPrep (Zymo Research, Irvine, CA, USA) according to the manufacturer’s instructions. RNA concentration and quality were determined by NanoDrop ND-1000 UV spectrophotometer (NaniDrop Technologies, Wilmington, DE, USA). 500 ng of total RNA was reverse transcript to cDNA by using RevertAid H Minus First Strand cDNA Synthesis Kit (Thermo Fisher Scientific, Waltham, MA, USA).

### Real-time quantitative PCR (qRT-PCR) analysis

The cDNA samples were amplified and applied by using iQ5 Real-time PCR Detection System (Bio-Rad, Hercules, CA, USA). The mRNA expression levels of the target genes were measured by ΔΔCt and normalized to glyceraldehyde-3-phosphate dehydrogenase (GAPDH). The qRT-PCR primer sequence were listed in Additional file 2: Table S1.

### Protein extraction

Cells were homogenized on ice using a homogenizer (LABSONIC® M ultrasonic homogenizer, Sartorius AG, Goettingen, Germany) with 60% amplitude and 0.6 cycle duration for 2 min using 12 mM sodium deoxycholate (Sigma-Aldrich, St Louis, MO, USA), 12 mM sodium lauroyl sarcosine (MP Biomedicals, Santa Ana, CA, USA), 50 mM triethylammonium bicarbonate (Sigma-Aldrich), protease inhibitor cocktail (BioShop, Burlington, Canada), and phosphatase inhibitor cocktail (Bionavas, Toronto, Ontario, Canada). Cell lysate was centrifuged at 16000×g for 20 min at 4 °C. Supernatants containing protein extract were then subject to the protein quantification using Pierce™ BCA Protein Assay Kit (Thermo Fisher Scientific).

### Reduction and alkylation

Protein extract was reduced with 10 mM dithiothreitol (BioShop) at 37 °C water bath for 30 min, and carboxymethylated with 55 mM iodoacetamide (Sigma-Aldrich) in the dark at room temperature for 30 min. Alkylated proteins were digested with Lys-C (1:100 w/w) (WAKO, Osaka, Japan) for 3 h and then digested with trypsin protease (1:100 w/w) (Thermo Fisher Scientific) overnight at 37 °C. The trypsin reaction was inactivated by acidified the peptide solution to a pH < 3 using trifluoroacetic acid (Sigma-Aldrich). Then, acidified peptide solution was combined with an equal volume of ethyl acetate (Sigma-Aldrich) and agitated vigorously for 1 min, followed by centrifugation at 15,700×g for 2 min to separate the aqueous and organic phases. The solution from the aqueous phase was dried using a centrifugal evaporator and then subjected to desalting using Styrenedivinylbenzene Empore disk membranes (SDB-XC) StageTips (#2340, 3 M, Nazarethm, Belgium) and eluted in a buffer containing 0.1% (v/v) TFA and 80% (v/v) acetonitrile (ACN) [27].

### Dimethyl labeling

300 μg of digested peptide were dried under centrifugal evaporator, then reconstituted in 100 mM TEAB. siRNA Control peptides was labeled with 4% (v/v) formaldehyde-H2 (Sigma-Aldrich), and siRNA ZNF322A peptides was labeled with 4% (v/v) formaldehyde-D2 (Sigma-Aldrich). 12 μL of 0.6 M sodium cyanoborohydride (Sigma-Aldrich) was added to each sample and incubated for 1 h at room temperature. Dimethyl labeling was inactivated by adding 48 μL of 0.1% (v/v) ammonium hydroxide (WAKO) on ice, and acidified by adding 30 μL of 10% (v/v) formic acid. The H2-labeled control was combined with the D2 labeled ZNF322A-silenced samples at 1:1 ratio.

### Phosphopeptide enrichment and fractionation

The phosphopeptide was enriched by aliphatic hydroxy acid-modified metal oxide chromatography (HAMMOC), where the home-made lactic acid-modified titania MOC tips were prepared [28]. The lactic acid-modified titania MOC tips were first equilibrated with Solution B (0.1% TFA, 80% ACN), and 300 mg/mL lactic acid. 100 μg of desalted peptide mixture was mixed with an equal volume of solution B and 300 mg/mL lactic acid, and loaded onto the lactic acid-modified titania MOC tips, washed with solution A (0.1% TFA and 5% ACN) followed by solution B. Phosphopeptides were eluted with 0.5 and 5% piperidine (Sigma-Aldrich), and the eluates were immediately acidified with 10% TFA and 20% phosphoric acid. For strong cation exchange peptide fractionation, phosphopeptides were eluted sequentially with buffers containing 0.1% TFA, 5% ACN, and in an ammonium acetate (WAKO) series [0, 20, 50, 100, or 500 mM]. The collected eluent were acidified with TFA and desalted. The samples were vacuum-dried, and then the phosphopeptides were resuspended in 0.5% TFA and analyzed by nanoLC–MS/MS.

### NanoLC–MS/MS analysis

NanoLC-MS/MS analysis was performed on a nanoACQUITY UPLC system (Waters, Milford, MA, USA) connected to an LTQ-Orbitrap XL hybrid mass spectrometer (Thermo Electron, Bremen, Germany) equipped with a nanospray interface (Proxeon, Odense, Denmark). Peptide samples were loaded onto a 2 cm × 180 μm capillary trap column and then separated in a 75 μm × 25 cm nanoACQUITY 1.7 μm BEH C18 column (Waters) at a flow rate of 300 nL/min. Mobile phase A consisted of 0.1% formic acid, and solution B consisted of 0.1% formic acid and 80% ACN. A linear gradient of 10 to 40% solution B in 90 min and 40 to 85% solution B in 10 min was employed throughout this study. Mass spectra from survey full scans were acquired on the Orbitrap (m/z 350–1500). The resolution was set to 60,000 at *m/z* 400 and the automatic gain control (AGC) was set to 1 × 10^6^ ions. The *m*/*z* values triggering MS/MS were put on an exclusion list for 90 s. The top ten most-intense precursor ions were selected from the MS scan for subsequent collision-induced dissociation MS/MS scan by ion trap (AGC target at 7000). Three biological replicates performed in two technical replicates.

### Data analysis

Raw MS spectra was processed for peak detection and quantitation by using MaxQuant software version 1.5.2.8 (http://maxquant.org). Peptide identification was performed by using the Andromeda search engine against the Swiss-PROT human database (December 9, 2015, reviewed). Search criteria used in this study were trypsin specificity, fixed modification of carbamidomethyl (C), variable modifications of oxidation (M), phosphorylation (STY), light dimethyl labeling (HC12HO), and heavy dimethyl labeling (D13CDO), and allowed for up to two missed cleavages [29]. A minimum of seven amino acids in the peptide length was required. The precursor mass tolerance was 3 ppm and the fragment ion tolerance was 0.5 Da. By using a decoy database strategy, peptide identification was accepted based on the posterior error probability with a false discovery rate of 1%. Precursor masses of already identified peptides were further searched and recalculated by using the “match between runs” option in MaxQuant. All the spectra and the related information were submitted to ProteomeXChange (http://www.proteomexchange.org/, Project accession PXD015936) and can be inspected by PRIDE Inspector.

### Functional and pathway enrichment analysis

Regulated proteins and phosphoproteins (normalized ratio ≥ 1.5 or ≤ 0.67) were annotated with GO terms. For the phosphoprotein with multiple phosphoryaltion sites, the largest expression changed of the phosphosite was used for the analysis. Over-represented GO terms were visualized as networks by Cytoscape version 3.2.1 [30]. Each node represents an enriched GO term (corrected *p*-value < 0.05), and the size of nodes represents the total number of genes in each GO term. Pie chart in a node represents the proportion of significantly regulated proteins derived from proteome and phosphoproteme (red: protein expression; yellow: protein phosphorylation). The edge represents gene overlap score between nodes over a given threshold (0.5). Pathway analysis was analyzed by REACTOME (v56, March 24, 2016).

### Kinase activity map analysis and protein-protein interaction network construction

Significantly regulated phosphoproteins were used for kinase activity map analysis by using DynaPho software [31] with the *p*-value < 0.05. The protein-protein interactions of differentially expressed proteins and phosphoproteins as well as predicted kinases were accessed by STRING database (version 10.0), https://string-db.org/, with the highest confidence (0.9). The protein-protein interaction network was depicted by Cytoscape version 3.2.1 [30]. Over-represented motifs were matched to known kinase motifs, and the position weight matrix (PWM) for each over-represented motif was generated based on motif-matching sequences from the results of Motif-X. PWM was constructed by counting the occurrence of each amino acid at each position and normalized at each position by the number of sequences. 300 kinase recognition motifs, resulting in 3656 high-quality kinase–substrate relationships, were obtained from the PhosphoNetworks database.

### Immunoblot analysis

Proteins were separated by 10% SDS-PAGE and transferred onto a polyvinylidene fluoride (PVDF) membrane (Millipore, Bedford, MA, USA). The membrane was blocked in 5% non-fat milk/PBST or 5% bovine serum albumin/PBST for 1 h, then incubated overnight with the following primary antibodies diluted in 5% non-fat milk/PBST or 5% bovine serum albumin/PBST at 4 °C: rabbit anti-ZNF322A (GTX121644, GeneTex, Irvine, CA 92606 USA), mouse anti-actin (MAB1501, Millipore, Billerica, Massachusetts, USA), rabbit anti-HSP27 S82 (MDBio, Taipei, Taiwan), rabbit anti-HSP27 (GTX101145, GeneTex), rabbit anti-eIF2α S51 (ab32157, Abcam, Cambridge, UK), rabbit anti-eIF2α (PA5–27366, Thermo Fisher Scientific), rabbit anti-IRS1 S1101 (A0446, Assay Biotech, Sunnyvale, CA, USA), rabbit anti-IRS1 (#2382, Cell signaling technology, Danvers, MA, USA), rabbit anti-AKT S473 (#9271, Cell signaling technology), rabbit anti-AKT (#9272, Cell signaling technology), rabbit anti-SQSTM1 (GTX100685, GeneTex), rabbit anti-LC3B/MAP1LC3B (NB6001384, Novus Biologicals, CO, USA) and mouse anti-actin (MAB1501, Millipore), rabbit anti-Pim-3 (#4165, Cell signaling technology), Rabbit anti-mTOR (GTX132803, GeneTex), Rabbit anti-mTOR S2448 (GTX101557, GeneTex). The membrane was then incubated with secondary HRP-conjugated anti-rabbit or anti-mouse IgG (Sigma-Aldrich) for 2 h at room temperature. Images were acquired using WesternBright ECL HRP substrate (Advansta, Menlo Park, CA, USA), UVP AutoChemi (UVP, Upland, CA, USA) and FluorChem M (ProteinSimple, San Jose, CA, USA).

### Glucose uptake assay

Glucose uptake was measured using a fluorescent non-metabolizable D-glucose analog 2-[*N*-(7-nitrobenz-2-oxa-1,3-diazol-4-yl) amino]-2-deoxy-D-glucose (2-NBDG; Cayman, Ann Arbor, Michigan, USA) [32]. 6 × 10^4^ A549 cells were seeded onto glass coverslips in 12-well plate for 24 h, then transfected with control siRNA and ZNF322A siRNA for 48 h. Cells were first pre-incubated in glucose-free and serum-free DMEM (Gibco Laboratories) for 15 min at 37 °C. Next, cells were incubated in 300 μM 2-NBDG for 6 h at 37 °C in the absence of glucose, and then washed 3 time with PBS and fixed with 3.7% paraformaldehyde (Sigma-Aldrich). The coverslips were mounted using AntiFade Prolong solution (Molecular Probes, Waltham, MA, USA) overnight. Images were acquired by using fluorescence microscope with Leica HCX FL PLAN 1006/1.25 oil objective, and SPOT Advanced software (Diagnostic Instruments, Sterling Heights, MI, USA). For the quantitative fluorescence microscopy analysis, images were analyzed based on the quantitative guideline in fluorescent microscopy imaging for accuracy and precision quantitation [33]. Randomly selected fields from siControl and siZN322A were analyzed to quantify fluorescence intensity and 2-NBDG molecules using ImageJ software (ImageJ, U.S. National Institutes of Health, Bethesda, Maryland, USA). The sample size determination was based on the statistical guideline, (*n* = 50) [34].

### Transmission electron microscope analysis

A549 lung cancer cell pellet were fixed in 2.5% glutaraldehyde in 0.1 M phosphate buffer (pH 7.4) overnight at 4 °C. Following three washes in 0.1 M phosphate buffer for 15 min. Sample was post-fixed with 1% osmium tetraoxide in a 0.1 M phosphate buffer for 90 min. The sample were then embedded in Spurr’s resin after dehydration in an acetone series overnight at 60 °C. Sections (~ 0.95 μm) were obtained with a glass knife and stained with 1% toluidine blue. Ultrasections (~ 95 nm) were cut and mounted on nickel or copper grids (75 or 100 mesh with 2.5% Formvar membrane), and then stained with 2% uranyl acetate for 20 min and washed. The sections were visualized by transmission electron microscope (H-7650, Hitachi-Science & Technology, Berkshire, United Kingdom).

## Results

### Quantitative phosphoproteomic profiling of A549 lung cancer cells regulated by ZNF322A

**Fig. 1:**
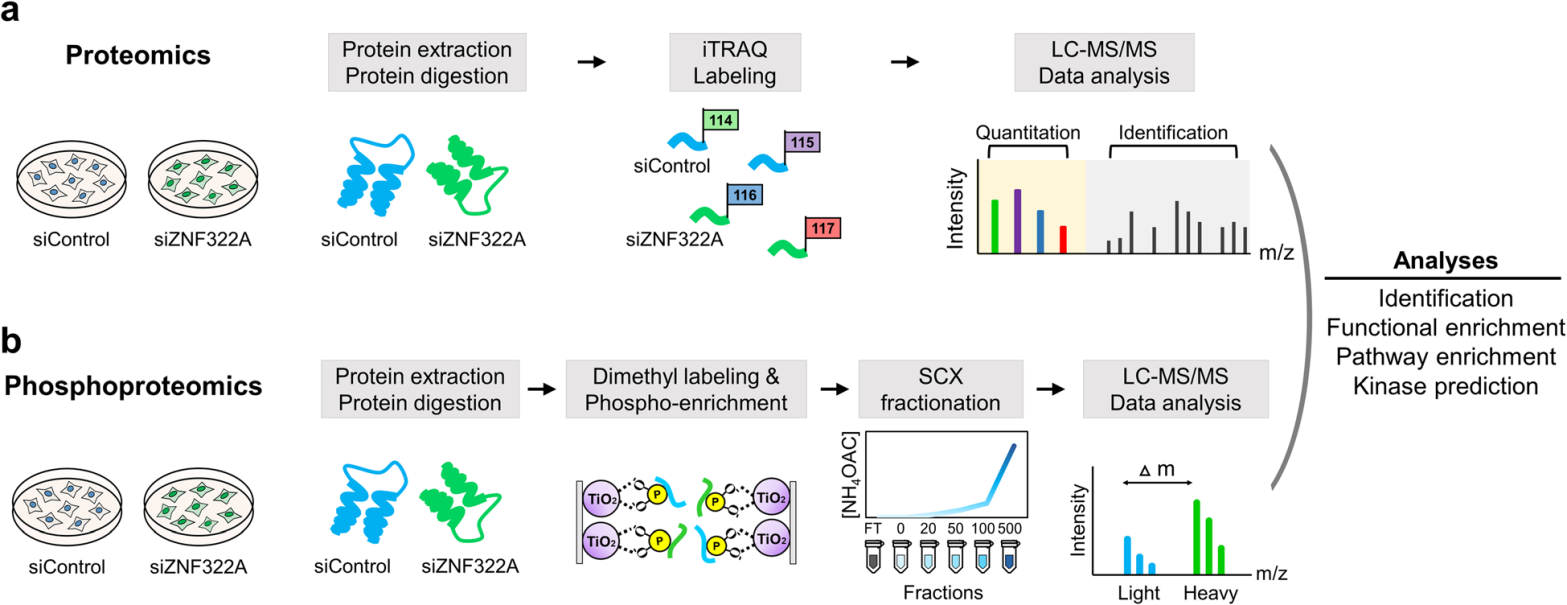
Overall workflow for the analyses of the proteome and phosphoproteome regulated by ZNF322A in lung cancer cells. **a** Experimental strategy for quantitative global proteomic profiling in response to silencing of ZNF322A (siZNF322A) in A549 cells. Protein extracts obtained from the transfected cells were digested, iTRAQ labeled, and analyzed with mass spectrometry. **b** Experimental strategy for quantitative phosphoproteomic profiling in response to siZNF322A in A549 cells. Protein extracts obtained from the transfected cells were digested, dimethyl labeled, phosphopeptide enriched, SCX fractionated, and analyzed with mass spectrometry. Construction of ZNF322A-perturbed functional network, and protein-protein interaction and the biological processes in lung cancer cells were validated by functional assays

**Fig. 2:**
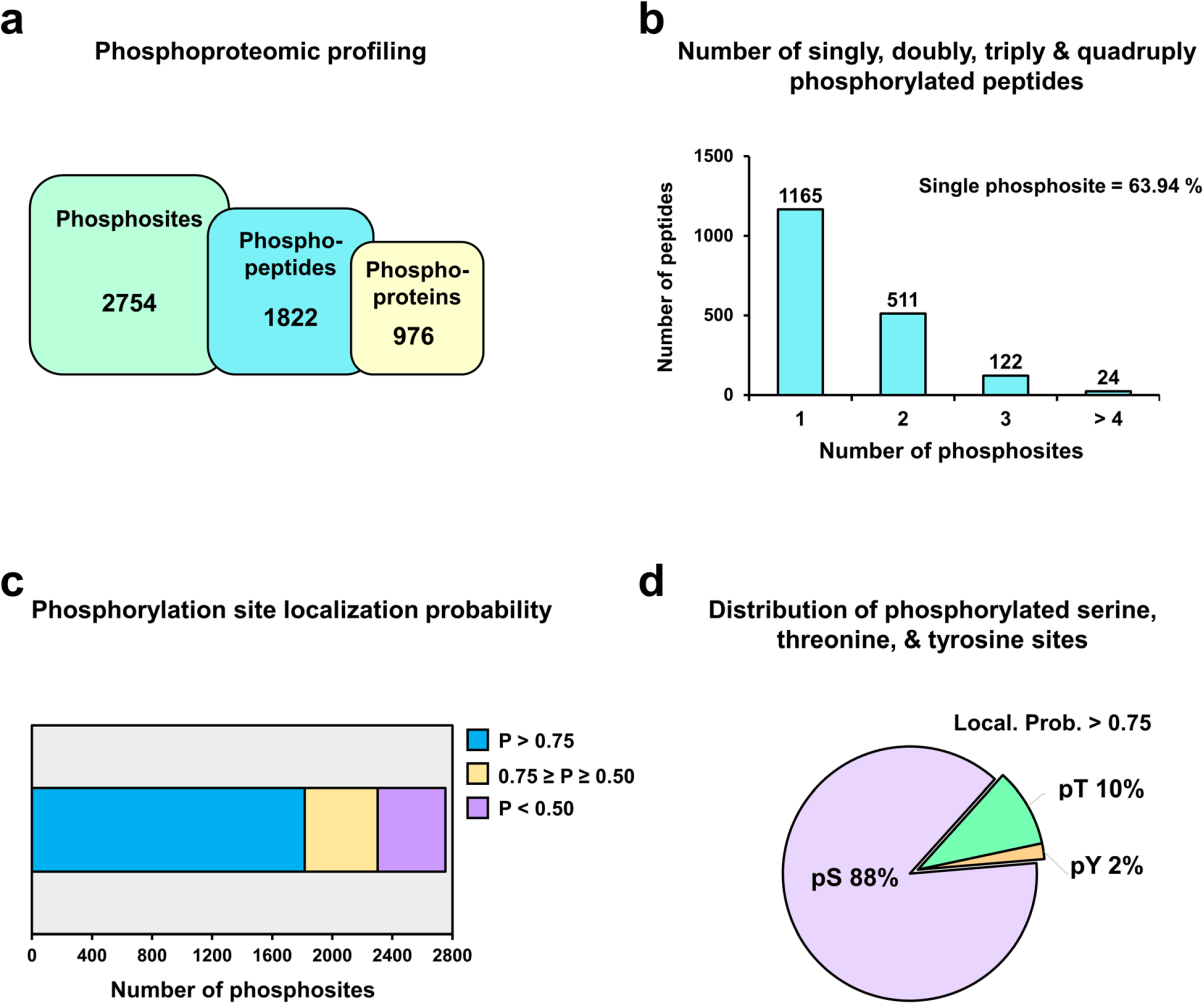
Phosphoproteomic profile of siZNF322A in A549 lung cancer cells. **a** Number of identified phosphoproteins, phosphopeptides, and phosphosites of the phosphoproteomics of ZNF322A-silenced A549 cells. **b** Number of singly, doubly, triply and quadruply phosphorylated peptides. **c** Localization probability of identified phosphorylated sites. Bar plot showed the percentages of localization probability, of which the majority of localization probability > 0.75, in ZNF322A-silencing phosphoproteome. **d** Distribution of phosphorylated serine, threonine, and tyrosine sites. Pie chart showed the distribution of phosphorylation sites on Ser (88%) Thr (10%) and Tyr (2%) residues

In our quantitative proteomic study of ZNF322A-silenced A549 lung cancer [7], we revealed that the majority of ZNF322A downstream proteins were involved in signal transduction and protein phosphorylation, thus, the ZNF322A-mediated phosphorylation network was established to better understand the mechanism and biological function of zinc-finger proteins in response to lung cancer. As depicted in Fig. 1a, samples were lysed, labeled with different iTRAQ-tags, then analyzed by LC-MS/MS for proteomic profiling in our previous study. For the phosphoproteomic profile, samples were lysed, labeled with stable-isotope dimethyl, phosphopeptide enriched and strong cation exchange (SCX) fractionated prior to LC-MS/MS analysis (Fig. 1b). To elucidate the regulatory network of ZNF322A in lung cancer cells, we analyzed the proteomic and phosphoproteomic profiles for biological function, pathway, and kinase activity using A549 lung cancer to explore the effect and roles of ZNF322A (Fig. 1). In total, we identified 2754 phosphosites on 1822 unique phosphopeptides and mapped to 976 phosphoproteins, with a phosphopeptide enrichment efficacy of 91.4% (Fig. 2a). Most peptides were singly or doubly phosphorylated, of which ~ 64% of peptides were singly phosphorylated (Fig. 2b). The phosphosites were divided into three categories based on their localization probability (P): class I (*P* > 0.75), class II (0.75 ≥ *P* ≥ 0.5), and class III (*P* < 0.5). Among the 2754 phosphosites, 1817 (65.97%) phosphosites were assigned with a probability of at least 0.75 (Fig. 2c). Also, the Class I phosphosites revealed a distribution of 88% phosphoserine (pSer), 10% phosphothreonine (pThr), and 2% phosphotyrosine residues (pTyr) (Fig. 2d). To assess an insight of ZNF322A-silencing phosphoproteomic profile, the quantified phosphosites ratio with high localization probability (Class I), were depicted in histogram of log_2_ transformed ratios (Additional file 1: Fig. S2). The distributions of phosphosite ratios follow normal distribution with the mean equals to − 0.02 and the standard deviation equals to 0.41. Phosphosites that showed 1.5-fold or greater changes in siZNF322A cells were considered to be up-regulated phosphosites, while those less than 0.67-fold changes were considered to be down-regulated phosphosites. Our phosphoproteomics quantified 45 up-regulated phosphosites and 72 down-regulated phosphosites corresponding to 42 and 61 phosphoproteins, respectively (Additional file 2: Table S2).

### Functional networks of ZNF322A-mediated proteins and protein phosphorylation

**Fig. 3:**
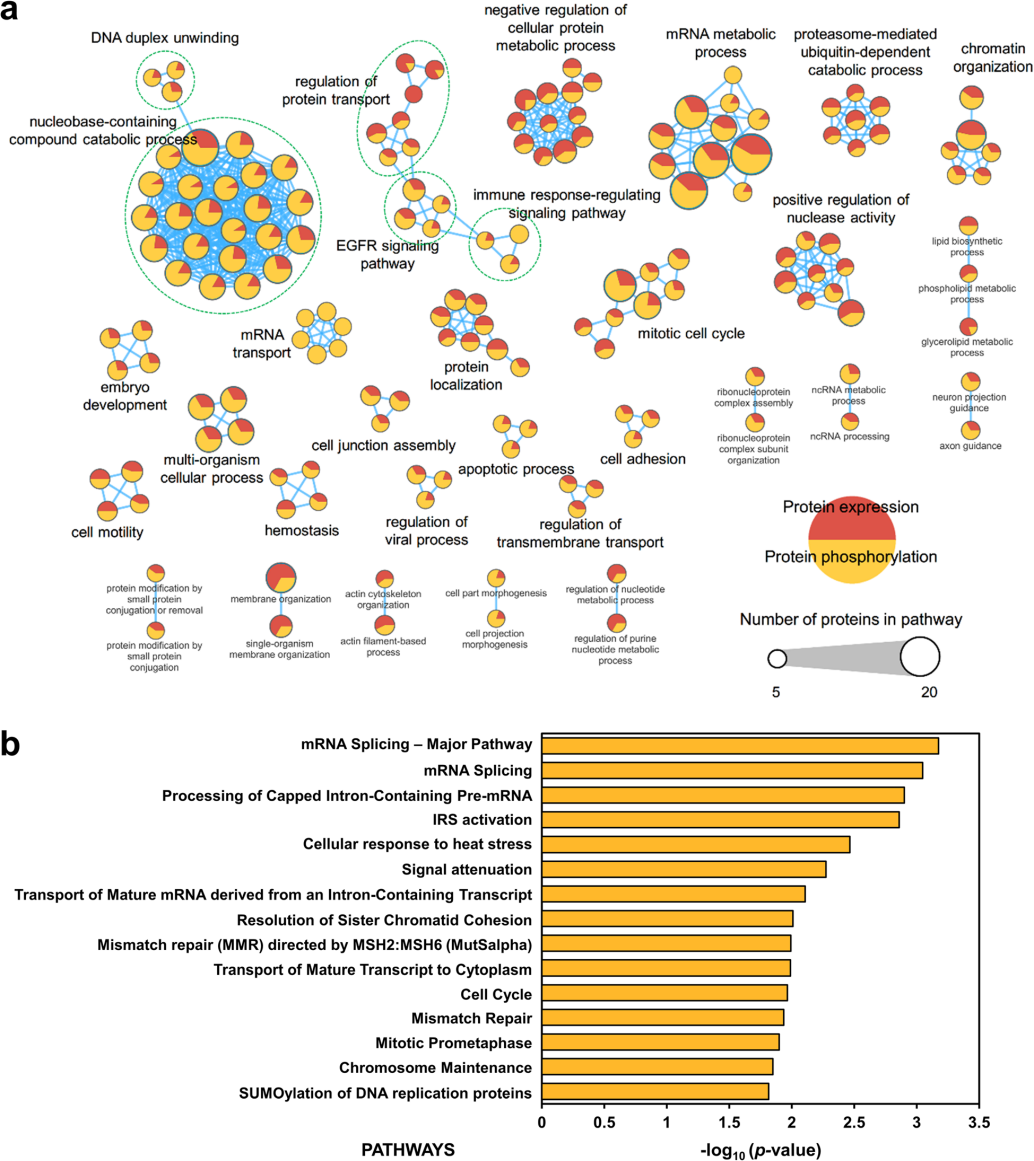
ZNF322A-mediated biological processes in A549 lung cancer cells. **a** Differentially regulated proteins from proteome and phosphoproteome profiles (normalized ratio ≥ 1.5 or ≤ 0.67) were annotated with GO terms. Each node represents an enriched GO term (corrected *p*-value < 0.05), and the size of nodes represents the total number of genes in each GO term. Pie chart in a node represents the proportion of significantly regulated proteins derived from proteomics and phosphoprotemics (red: protein expression; orange: protein phosphorylation). The edge represents gene overlap score between nodes over a given threshold (0.5). Functionally related GO terms are manually grouped and labeled. **b** Pathway analysis of ZNF322A-regulated phosphoproteins. Top 15 enriched pathways of the phosphoproteomics with the lowest *p*-value. The number of entities is shown as bar label

To establish the functional roles of ZNF322A in lung cancer, we analyzed phosphoproteomic and our previous proteomic studies [7] to perform Gene Ontology (GO) and pathway enrichment. To reveal ZNF322A-mediated biological processes in lung cancer, we performed GO enrichment analyses of 77 proteins and 103 phosphoproteins that exhibited significantly changed in proteomics and phosphoproteomics under ZNF322A silencing (Fig. 3a). Consistent to our previous ZNF322A silencing proteomic profile and studies that ZNF322A is related to the regulation of nuclease activity, embryonic development, cell cycle, cell motility, and apoptosis, suggesting ZNF322A-regulated protein phosphorylation and signal transduction also play a pivotal role in lung cancer progression (Fig. 3a, Additional file 2: Table S3). Next, we investigated the signaling pathways specifically triggered or modulated by ZNF322A at the protein phosphorylation level, the differentially phosphorylated proteins were employed in pathway enrichment analysis, apparently the top 15 enriched pathways were mostly related to RNA processing, chromosome organization and maintenance, and cell cycle (Fig. 3b, Additional file 2: Table S4). Interestingly, IRS activation, cellular response to heat stress, and signal attenuation arose our further interest to investigate the association to ZNF322A.

### ZNF322A modulates glucose uptake via the IRS1/PI3K/AKT pathway in lung cancer cells

**Fig. 4:**
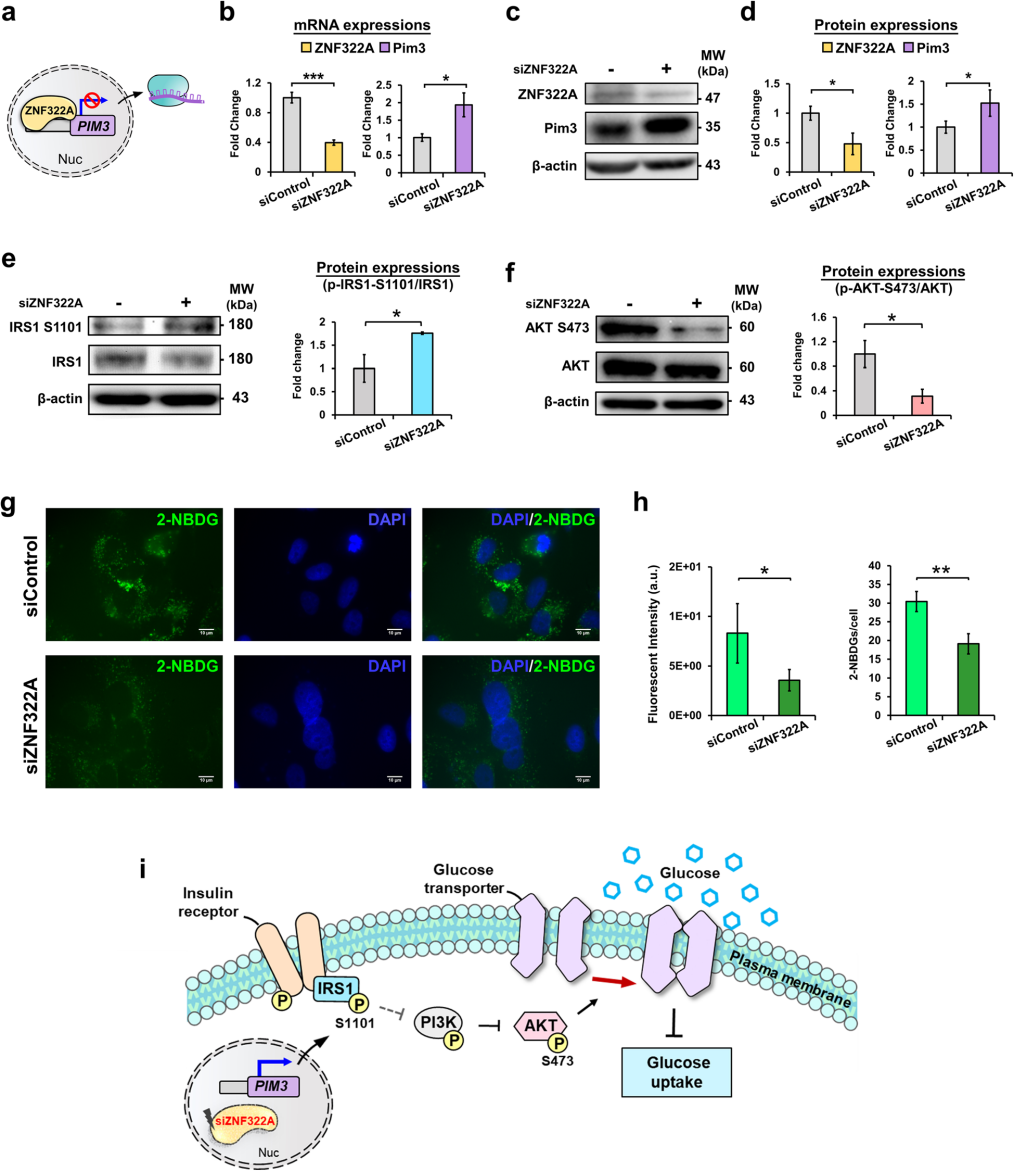
ZNF322A modulates glucose uptake via the IRS1/PI3K/AKT pathway in A549 lung cancer cells. **a** Schematic representation of the inhibitory transcription regulation of ZNF322A on PIM3. **b** Relative mRNA levels of ZNF322A and PIM3 were examined by RT-qPCR analysis, and normalized by GAPDH. **c-d** Proteins were extracted from ZNF322A-silenced (siZNF322A) A549 cells after 48 h transfection. Protein expressions of ZNF322A and PIM3 were examined by immunoblot analysis. Representative western blot (c) and associated densitometric analysis (d) for ZNF322A and PIM3 expressions in A549 lung cancer cells. **e-f** Protein expressions of IRS1^S1101^ and AKT ^S473^ phosphorylation were examined by immunoblot analysis. Phosphorylation levels were determined and normalized to the level of total proteins. The normalized values were compared between siRNA control (siControl) and siZNF322A groups. **g-h** Effect of 2-NBDG uptake upon ZNF322A perturbation in A549 lung cancer cells. Representative fluorescence microscopy images of 2-NBDG uptake ability in siRNA control (upper) and siZNF322A-silenced (bottom) A549 cells. 2-NBDG: green; DAPI: blue; scale bar: 10 μm (g). Mean fluorescence intensity (left) and number (right) of 2-NBDG in siControl and siZNF322A cells (h). (**i**) Schematic representation of the depletion of ZNF322A by siRNAs in A549 lung cancer cells transcriptionally regulates PIM3 kinase to induces IRS1^Ser1101^ phosphorylation, which attenuates PI3K/AKT signaling pathway and inhibits AKT^S473^, leading to glucose uptake blockade. β-actin is the internal control to normalize protein expression. The bars represent densitometric analysis of three biological replicates and the data are shown as mean ± SD. * *p* < 0.05; ** *p* < 0.01; *** *p* < 0.001

To elucidate the ZNF322A-regulated phosphorylated protein network in response to insulin activation (Fig. 3b), we analyzed our previous anti-ZNF322A ChIP-seq data [9], and found the upstream kinase, serine/threonine-protein kinase pim-3 (PIM3), of Insulin receptor substrate 1 (IRS1) had ZNF322A binding signals [35]. As ZNF322A has been suggested to transcriptionally regulate expression of PIM3, qRT-PCR and immunoblotting were performed on A549 lung cancer cells with ZNF322A perturbation (Fig. 4a). Silencing of ZNF322A significantly upregulated the mRNA (Fig. 4b) and protein (Fig. 4c and d) expressions of PIM3. These results suggest the inhibitory transcriptional regulation of ZNF322A on PIM3 in A549 lung cancer cells. Moreover, ZNF322A binding signals were found to be located at IRS1 promotor in anti-ZNF322A ChIP-seq profile [9], suggesting a transcriptionally regulation of ZNF322A on IRS1 (Additional file 1: Fig. S3). IRS1 is one of the key mediator for glucose metabolism that regulates translocation of glucose transporter through the PI3K/AKT signaling pathway to facilitate glucose uptake [19, 36–38]. Phosphorylation of IRS1 at serine^1101^ participates in suppressing insulin activation via inhibition of PI3K/AKT signaling [39]. Based on the above findings, we identified a phosphorylation residue, serine^1101^, in IRS1 with a 1.54-fold changed upon ZNF322A silencing of A549 lung cancer cells in the quantitative phosphoproteomic data (Additional file 2: Table S2). To ascertain the stimulatory effect of ZNF322A on lung cancer in the IRS1/PI3K/AKT pathway, we further analyzed IRS1 and AKT expressions of ZNF322A-silenced A549 cells at 48 h post-transfection by immunoblotting (Fig. 4e). The quantified results showed that IRS1 phosphorylation at serine^1101^ was significantly upregulated, and the phosphorylation of serine^473^ in AKT was significantly downregulated in ZNF322A-silencing lung cancer cells (Fig. 4f). Since the IRS1/PI3K/AKT signaling pathway participates in the process of glucose transport into cells, we further examined glucose uptake by a fluorescent glucose analog, 2-NBDG, to determine the effect of ZNF322A in lung cancer. 2-NBDG fluorescent levels were monitored by fluorescence microscopy after transfection with siZNF322A for 48 h (Fig. 4g). The fluorescent intensity and number of 2-NBDG uptake were significantly decreased in ZNF322A-silenced cells (Fig. 4h). Taken together, we purpose that the depletion of ZNF322A in A549 lung cancer cells transcriptionally regulates PIM3 kinase to induce IRS1^Ser1101^ phosphorylation, which attenuates PI3K/AKT signaling pathway and inhibits AKT^S473^, leading to glucose uptake blockade (Fig. 4i). This is the first time to discover that silencing of ZNF322A is capable to mediate the regulation of insulin signal transduction pathway and caused glucose starvation in lung cancer cells.

### ZNF322A mediates heat shock protein 27 phosphorylation and elicits unfolded protein response

**Fig. 5:**
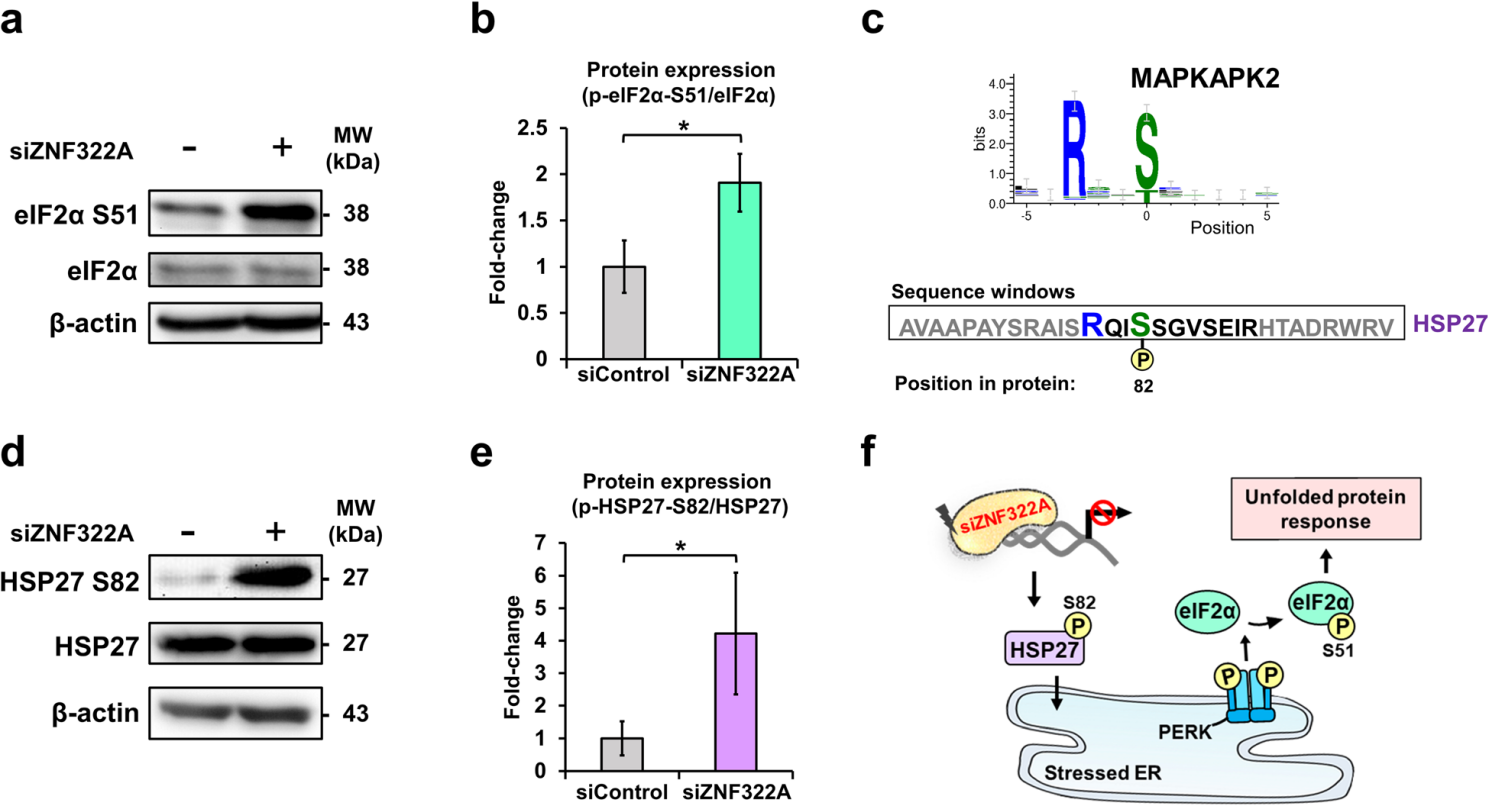
ZNF322A mediates heat shock protein 27 phosphorylation and elicits unfolded protein response. **a** The protein expressions of eIF2α in UPR signaling pathway were analysis by immnoblotting. **b** Phosphorylation of eIF2α^S51^ was determined and normalized to the level of total eIF2α. The normalized values were then compared between siControl and siZNF322A. **c** Enriched consensus motifs of MAPKAPK2 on HSP27^S82^ were predicted and identified in ZNF322A-silenced phosphoproteome. MS/MS spectrum for HSP27 (QIpSSGVSEIR), and fragment ions in the MS/MS spectrum localize at serine^82^ in HSP27. **d** Validation of HSPB1^S82^ phosphorylation using immunoblot analysis. **e** Phosphorylation of HSP27 was determined and normalized to the level of total HSP27. The normalized values were compared between siControl and siZNF322A groups. **f** Schematic representation of the heat stress responses of HSP27^S82^ via UPR pathway by silencing of ZNF322A in A549 lung cancer. β-actin is the internal control to normalize protein expression. The bars represent densitometric analysis of three biological replicates and the data are shown as mean ± SD. * *p* < 0.05

We next sought to determine whether the ZNF322A is linked to cellular response to heat stress in lung cancer (Fig. 3b). Heat stress causes the accumulation of misfolded proteins in the endoplasmic reticulum (ER), which leads to activation of the unfolded protein response (UPR), as phosphorylation of eIF2α at serine^51^ indicates that UPR is triggered to attenuate protein synthesis [40]. The perturbation of UPR was validated by protein phosphorylation of serine^51^ on eIF2α which were increased by 1.9-fold changed upon ZNF322A silencing (Fig. 5a and b). To investigate the regulatory network of ZNF322A, we analyzed the significantly regulated proteins, phosphoproteins, and predicted kinases into a protein-phosphoprotein-kinase interaction (PPKI) network (Additional file 1: Fig. S4). The kinase activity analysis provided us a robust path to predict the complicated kinase-substrate relationship, and a total of eighty-four kinases were enriched using DynaPho [31]. HSP27 is a substrate of MAPK-activated protein kinase 2 (MAPKAPK2), participating in p38 MAPK pathway [41]. MAPKAPK2 is the predicted kinase to the sequence window of HSP27 mass spectrum, which recognized a short linear sequence of amino acids with overrepresented motif patterns for initiating phosphorylation at HSP27 serine^82^ (Fig. 5c). Thus, we further ascertained our mass spectrometric observation with immunoblot analysis using a phospho-specific antibody against serine^82^ on HSP27 (Fig. 5d). The immnuoblot results showed a significantly increased of HSP27 at serine^82^ with a 4.2-fold changed at 48 h siZNF322A in lung cancer (Fig. 5e). The intracellular concentration of the HSP27 increases after heat and metabolic stresses [42]. Besides, HSP27 is involved in ER stress and capable to bind to improperly folded proteins and further transfer them into protein degradation machinery such as autophagosomes [43, 44]. The above evidence and findings suggest that knockdown of ZNF322A may suppress lung cancer proliferation through HSP27 at serine^82^ phosphorylation, and induces UPR (Fig. 5f).

### Silencing of ZNF322A induces autophagosome formation in lung cancer cells

**Fig. 6:**
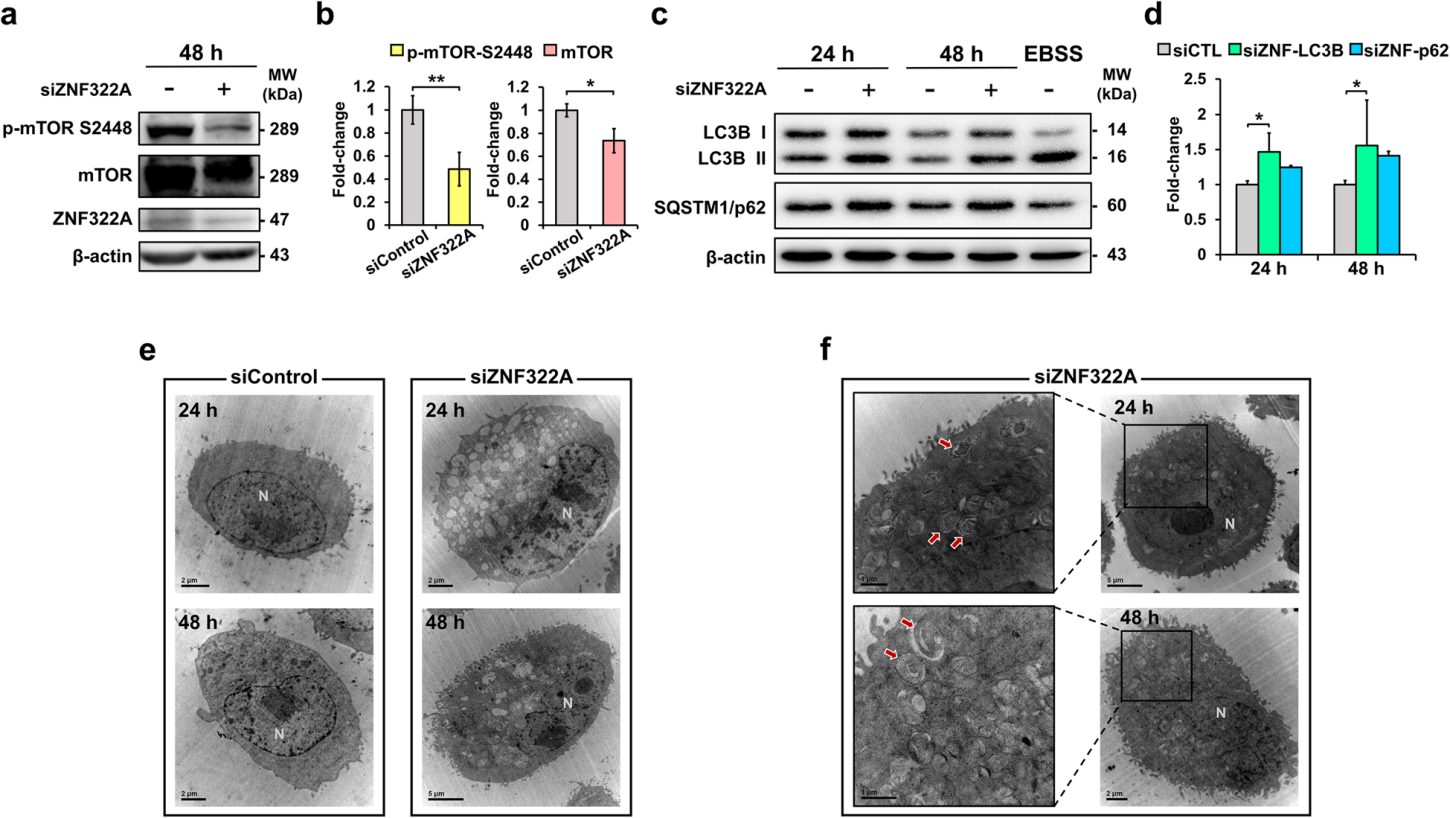
Silencing of ZNF322A induces autophagosome formation in lung cancer cells. **a**-**b** Protein expression of mTOR and p-mTOR^S2448^ was determined and normalized by immunoblotting. **c**-**d** Induction of autophagy related proteins including LC3B-I, LC3B-II, and SQSTM1 were analyzed and quantified by immunoblotting. Earle’s balanced salts solution (EBSS) was used as positive control to induce autophagy. **e**-**f** Electron microscopy of A549 lung cancer cells after 24 or 48 h of siControl or ZNF322A siRNA transfection was analyzed. Double- and multi-membrane autophagosomes were clearly observed in ZNF322A-silenced cells. Final stages of autophagic cell death were accompanied by ballooning of the perinuclear space and disappearance of cellular organelles such as autophagosomes, autolysosomes, and ER. Arrows: diverse autophagosomes, N: nuclear. β-actin is the internal control to normalize protein expression. The bars represent densitometric analysis of three biological replicates and the data are shown as mean ± SD. * *p* < 0.05; ** *p* < 0.01

ZNF322A modulated the IRIS/PI3K/AKT and HSP27/MAPK pathways to perturb glucose uptake and UPR upon ZNF322A-slienced A549 lung cancer cells, and the cumulative evidence also suggest that suppression of the PI3K/AKT/mTOR pathway and induction of HSP27-regulated misfolded proteins triggers cytoprotective autophagy in cancer [44–47], hence, we further investigated whether autophagy was mediated by ZNF322A in lung cancer. First, we assessed mTOR phosphorylation to determine mTOR activity upon ZNF322A-silencing A549 cells, and found phosphorylation of serine^2448^ in mTOR and mTOR significantly decreased by siZNF322A (Fig. 6a and b). The results revealed that silencing of ZNF322A represses mTOR activity, thereby promoting autophagy. Next, we determined the expression levels of autophagy-related proteins including microtubule-associated protein 1 light chain-3B (LC3B) and sequestosome 1 (SQSTM1/p62) after 24 and 48 h under ZNF322A silencing (Fig. 6c), and found that the ratios of LC3B11/LC3B I and SQSTM1 were significantly increased, suggesting the inhibition of ZNF322A may facilitate cellular autophagy in lung cancer (Fig. 6d). Consistently, transmission electron microscopy (TEM) images of A549 lung cancer cells clearly showed the formation of double and multilayered membrane-bounded structures, which signify autophagosomes were formed in the siZNF322A compared to the siControl at 24 and 48 h transfection (Fig. 6e). Autophagy processes start with the formation of double- and multi-membrane autophagosome, and mature and fuse with lysosome to generate autolysosome. The morphological features of autophagic vacuoles manifested cell death figures and typically increased autophagosomes and autolysosomes numbers, as well as rupture and blebbing of plasma membranes were observed in the siZNF322A cells in our study (Fig. 6f). mTOR is a key component that coordinately regulates the balance between cell growth and autophagy in response to cellular physiological conditions and environmental stress. Upon stress, mTOR is inhibited, leading to the induction of autophagy in cells [48]. Taken together, we observed autophagic vacuolization, which is commonly observed in cells undergoing apoptotic or necrotic cell death, this observation hints that cell death under silencing of ZNF223A associates with autophagy mechanism via IRIS/PI3K/AKT and HSP27/MAPK pathways.

## Discussion

ZNF proteins, as a huge gene family, exert distinct functions in several aspects of cellular processes, including differentiation, proliferation, apoptosis and metastasis [2–4]. Overexpression of ZNF322A promoted tumor growth and metastasis in both cell-based and animal-based studies, moreover, ZNF322A was significantly overexpressed in lung cancer patients and associated with poor overall survival, suggesting ZNF322A is an oncogene in lung cancer [7, 8]. Our previous studies have been using proteomic approach to investigate the regulated proteins in different network and disease. Consistent with our previous reports, RNA processing and RNA splicing were observed to be involved in the ZNF322A downstream signaling pathway, and the functional enrichment also identified other processes such as regulation of protein transport, hemostasis, and metabolic process by analysis the differentially regulated phosphoproteins and proteins individually.

In the REACTOME pathway enrichment analysis, IRS activation was discovered as an induced pathway upon ZNF322A silencing. Insulin receptor substrate 1 (IRS1) and growth factor receptor-bound protein 10 (GRB10) were identified in our phosphoproteomic results, which are participated in IRS activation. IRS1 is a cytoplasmic signaling adapter protein that is able to integrate different signaling cascades by activation through cell surface receptors, including the insulin, insulin-like growth factor 1 (IGF-1), growth hormone (GH) receptors [49]. Phosphorylation of IRS1 on tyrosine residues is required for insulin-stimulated responses, while phosphorylation of IRS1 on serine residues has a dual role, either to enhance or to terminate the insulin effects [50, 51]. Several epithelial tumors depend constitutive activation of this pathway to increase their glucose supply [32]. IRS1^S1101^ is phosphorylated by protein kinase C theta (PKCθ), Pim and ribosomal protein S6 kinase beta-1 (RPS6KB1) [32, 35, 39, 52]. PKCθ phosphorylates serine residues on IRS1, which dissociates from the insulin receptor, leading to decreased signaling via PI3K/AKT and reduced glucose uptake. On the other hand, GRB10 also disrupts the association of IRS1 with insulin receptor and further inhibit PI3K/AKT pathway [53]. GRB10^S104^ was also identified upregulated in ZNF322A-silenced cells, but the phosphosite has not yet been annotated. In our study, we suggested that knock-down of ZNF322A in lung cancer induces phosphorylation of IR1S that attenuates PI3K/AKT/mTOR signaling pathway, leading to glucose starvation which may trigger autophagosome formation. Moreover, we also assessed the phosphorylation level of IRS1 at serine^1101^ in H1299 cell line to establish the effect of ZNF322A on NSCLC. Phosphorylation of IRS1^S1101^ increased in ZNF322A-silenced H1299 lung cancer cells which suggests ZNF322A is a regulator for IRS1/AKT/mTOR signaling pathway in NSCLC (Additional file 1: Fig. S5).

On the other hand, HSP27 is a 27 kDa molecular chaperone belongs to small heat shock family which is responsible for ER stress and cellular response to heat stress [42, 54–56]. Phosphorylation of HSP27 was found to be contributed to inhibit cancer cell growth due to its conformational changes, leading complex dissociation, and subsequent loss of chaperone activity [57–59]. Consistent with our findings, phosphorylation of HSP27 at serine^82^ was identified in siZNF322A A549 cells from our quantitative phosphoproteomics. In addition, we also validated the phosphorylation of HSP27 at serine^82^ in H1299 cell line to establish the effect of ZNF322A on NSCLC. Phosphorylation of HSP27^S82^ increased in ZNF322A-silenced H1299 lung cancer cells which indicates that ZNF322A may trigger the HSP27/MAPK signaling pathway in NSCLC (Additional file 1: Fig. S5). In our phosphoproteomic profile, phosphorylation of heat shock protein HSP 90-alpha (HSP90AA1) at serine^231^ was also identified with 11.06 fold-changed upon silencing of ZNF322A, which may contribute to autophagy, however the phosphosite-specific antibody has not yet been established. As previously reported, ZNF322A may act as regulator in gene transcription mediated by the MAPK signaling pathways [60]. Consistent with our findings that siZNF322A upregulated the phosphorylation of HSP27 and MAPKAPK2, participating in p38 MAPK pathway which is responsible for the autophagosome formation.

Collectively, we proposed glucose starvation and UPR in siZNF322A lung cancer cells altered mTOR signaling pathway and further induced autophagosome formation. In the current studies, mTOR has emerged as a critical effector commonly deregulated in human cancer, especially in NSCLC [61]. mTOR pathway is a member of the PI3K cell survival pathway and plays an important role in the regulation of cell growth and proliferation [62]. Another crucial role of mTOR pathway is the AKT, which is activated downstream of growth factor-stimulated PI3K activity and phosphorylates a variety of upstream and downstream substrates. Recently, we demonstrated a novel oncogenic regulatory mechanism of phosphorylation by AKT in regulating ZNF322A protein stability and transcriptional activity that promote lung cancer progression [63]. Autophagy serves as a mechanism of tumor suppression, and as an adaptive stress response in tumor cells to maintain their survival [24, 64], which is a self-destructing homeostatic process that removes damaged organelles, restrained cell growth and non-apoptotic cell death [21]. Studies in the past decades have shown that autophagy can also induce cell death, which occurs in mammalian cells and tissues in response to pathophysiological stimuli [65].

## Conclusion

**Fig. 7:**
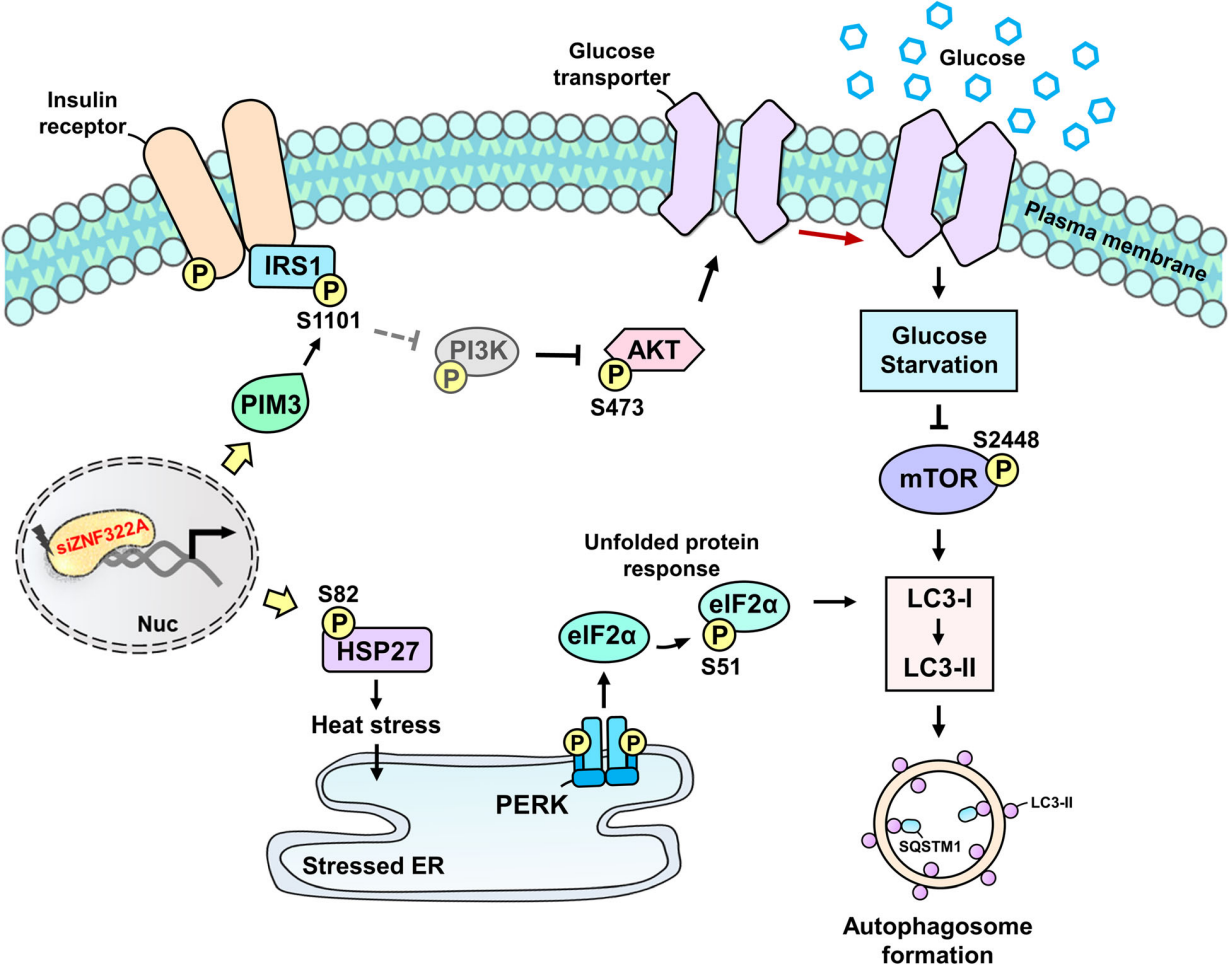
Silencing of ZNF322A was attributed to its induction of autophagy via protein phosphorylation. Silencing of ZNF322A transcriptionally regulates PIM3 to phosphorylate IRS1^S1101^ and further inhibits the PI3K/AKT/mTOR pathways, limiting access of lung cancer cell to glucose. Silencing of ZNF322A induces phosphorylation of HSP27^S82^ and leads to activation of the unfolded protein response (UPR). Glucose starvation and heat stress-elicited activation trigger autophagosome formation in ZNF322A-silenced A549 lung cancer cells

ZNF322A is determined to be an oncogene in lung cancer [66], however, the modulatory role of ZNF322A remained to be clarified. Taking advantage of high-throughput HAMMOC phosphopeptide enrichment and mass spectrometry technology, we are able to profile proteomic and phosphoproteomic data in order to elucidate the ZNF322A-regulated downstream phosphoproteins using integrative analyses. In this study, the two proteomic profiles were generated in different timeframe, which may cause a low overlapping of two proteome datasets. Thus, the differentially regulated proteins of both datasets were used for the analysis of pathway enrichment and PPKI network to establish the pivotal roles of ZNF322A in the protein and protein phosphorylation levels. Variety of biological functions and ZNF322A-mediated kinase-substrate phosphorylation network were revealed in this study. Remarkably, IRS1^S1101^ and HSP27^S82^ were significantly increased, which was considered as the key downstream targets of ZNF322A. Using bioinformatics approaches including functional enrichment, network analysis and phosphorylation motif analysis, we further showed the involvement of mTOR signaling pathway, and demonstrated that ZNF322A silencing in lung cancer cells induced autophagic cell death, but more underlying mechanism requires detailed investigation. The overall schematic mechanism is illustrated in Fig. 7 and indicates that ZNF322A-silenced A549 lung cancer cells trigger autophagy by glucose starvation and UPR through phosphorylation of IRS1^S1101^ and HSP27^S82^ to inhibit PI3K/AKT/mTOR pathways and to elicit heat stress. Our results not only provide useful information for phosphoproteomic research in ZNF322A but also reveal a new picture of signal transduction corresponding to ZNF322A regulation, and provides insights for further investigation of protein phosphorylation status in ZNF322A-mediated lung cancer. Overall, our data suggest that therapeutic strategies such as efficient delivery of ZNF322A interference RNAs or treatment of targeted inhibitors of ZNF322A downstream post-transcriptional modification can be a potential molecular therapeutic method for lung cancer in the future.

## Supplementary information

**Additional file 1: Figure S1.** mRNA and protein expressions of ZNF322A in ZNF322A silenced A549 lung cancer cells. **Figure S2.** Distribution of phosphorylated site ratios. **Figure S3.** Snapshot of the ChIP-seq binding profile of ZNF322A at IRS1 gene in lung cancer cell. **Figure S4.** Analysis of protein-phosphoprotein-kinase interactions in ZNF322A silenced A549 lung cancer cells. **Figure S5.** IRS1^S1101^ and HSP27^S82^ phosphorylation in ZNF322A silenced H1299 lung cancer.

**Additional file 2: Table S1.** Sequence of primers and annealing conditions for real-time quantitative PCR. **Table S2.** The list of significantly regulated phosphosites of siZNF322A in A549 lung cancer cells. **Table S3.** Gene list of Gene Ontology analysis of siZNF322A in A549 lung cancer cells. **Table S4.** Signaling pathway enrichment analysis of siZNF322A phosphoproteomics in A549 lung cancer cells by REACTOME.

## Data Availability

The raw data supporting this article is available in the ProteomeXchange repository, Project accession: PXD015936; http://www.ebi.ac.uk/pride.

## Abbreviations

2-NBDG: 2-(N-(7-nitrobenz-2-oxa-1,3-diazol-4-yl)amino)-2-deoxyglucose
Add1: Alpha-adducin
AKT: Protein kinase B
C2H2: Cysteine-2/histidine-2
CCND1: Cyclin D1
CTND1: Catenin delta-1
HAMMOC: Hydroxy acid-modified metal oxide chromatography
HSP27: Heat shock protein beta-1
IRS1: Insulin receptor substrate 1
iTRAQ: Isobaric tag for relative and absolute quantitation
LC-MS/MS: Liquid chromatography-tandem mass spectrometry
MAPKs: Mitogen-activated protein kinases
MAPKAPK2: Mitogen-activated protein kinase-activated protein kinase 2
mTOR: Mammalian target of rapamycin
EGFR: Epidermal growth factor receptor
eIF2α: Eukaryotic translation initiator factor 2α
ER: Endoplasmic reticulum
ERK: Extracellular signal-regulated kinase
NSCLC: Non-small cell lung cancer
GO: Gene Ontology
JIP4: c-Jun-amino-terminal kinase-interacting protein 4
JNK: c-jun NH2-terminal kinase
nanoLC-MS/MS: Nanoscale liquid chromatography–tandem MS
PI3K: Phosphoinositide 3-kinase
PIM3: Serine/threonine-protein kinase pim-3
PPI: Protein-protein interaction
pSer/pS: Phosphoserine
pThr/pT: Phosphothreonine
pTyr/pY: Phosphotyrosine
PWM: Position weight matrix
PTM: Post-transcriptional modification
qRT-PCR: Quantitative real time polymerase chain reaction
RPS6KB1: Ribosomal protein S6 kinase beta-1
SCX: Strong cation exchange
siRNA: Small interfering RNA
TEM: Transmission electron microscopy
UPR: Unfolded protein response
ZNF: Zinc finger
