# Supplementary material for: ZNF322A-mediated protein phosphorylation induces autophagosome formation through modulation of IRS1-AKT glucose uptake and HSP-elicited UPR in lung cancer
Source: J Biomed Sci. 2020 Jun 23;27:75. doi: 10.1186/s12929-020-00668-5 (PMC7310457; doi:10.1186/s12929-020-00668-5)
Supplement: Supplementary file 1 — Additional file 1: Figure S1. mRNA and protein expressions of ZNF322A in ZNF322A silenced A549 lung cancer cells. Figure S2. Distribution of phosphorylated site ratios. Figure S3. Snapshot of the ChIP-seq binding profile of ZNF322A at IRS1 gene in lung cancer cell. Figure S4. Analysis of protein-phosphoprotein-kinase interactions in ZNF322A silenced A549 lung cancer cells. Figure S5. IRS1S1101 and HSP27S82 phosphorylation in ZNF322A silenced H1299 lung cancer. [file 12929_2020_668_MOESM1_ESM.docx]

**Additional file 1: Supplementary Figures**

**ZNF322A-mediated protein phosphorylation induces autophagosome formation through modulation of IRS1-AKT glucose uptake and HSP-elicited UPR in lung cancer**

Chantal Hoi Yin Cheung^1^, Chia-Lang Hsu^1,2^, Tsai-Yu Lin^1^, Wei-Ting Chen^1^, Yi-Ching Wang^3,4^, Hsuan-Cheng Huang^5*^, and Hsueh-Fen Juan^1,6*^

^1^Department of Life Science, National Taiwan University, Taipei 10617, Taiwan.

^2^Department of Medical Research, National Taiwan University Hospital, Taipei 10002, Taiwan

^3^Department of Pharmacology, College of Medicine, National Cheng Kung University, Tainan 70101, Taiwan.

^4^Institute of Basic Medical Sciences, College of Medicine, National Cheng Kung University, Tainan 70101, Taiwan.

^5^Institute of Biomedical Informatics, National Yang-Ming University, Taipei 11221, Taiwan.

^6^Graduate Institute of Biomedical Electronics and Bioinformatics, National Taiwan University, Taipei 10617, Taiwan.


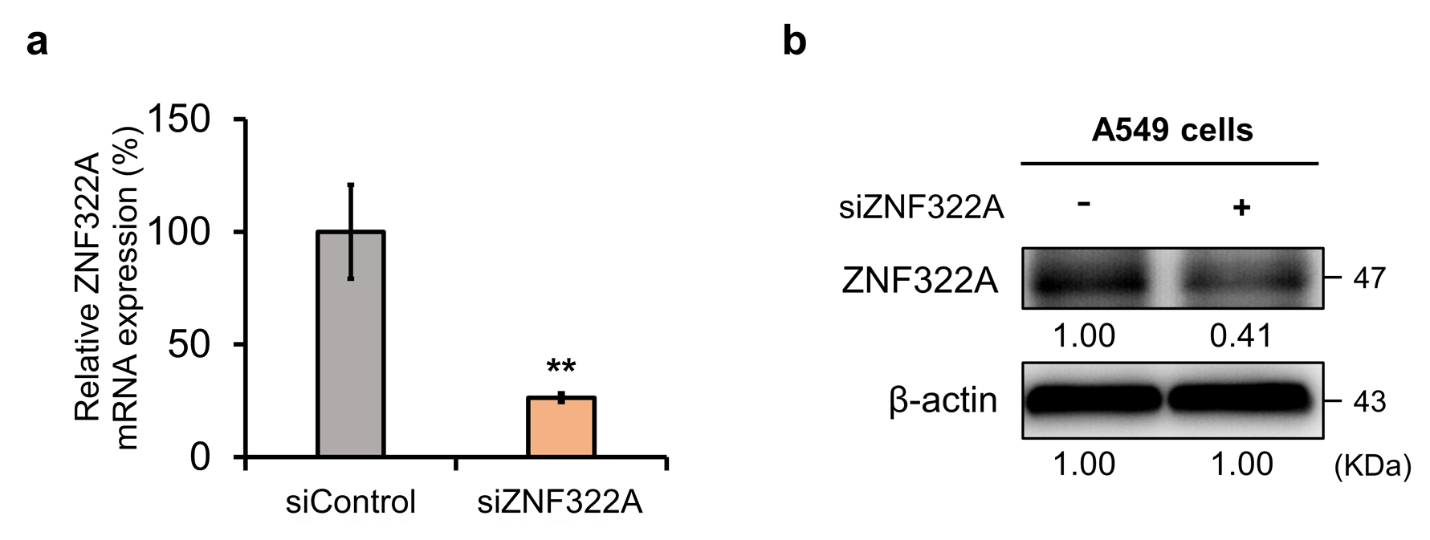


**Figure S1. mRNA and protein expressions of ZNF322A in ZNF322A silenced A549 lung cancer cells.** (a) Relative mRNA levels of ZNF322A were examined by RT-qPCR analysis, and normalized by GAPDH. Asterisks indicate signiﬁcant differences from the siControl. (b) Proteins were extracted from ZNF322A silenced A549 cells after 48 h transfection, and the ZNF322A protein expressions were examined by western blot analysis. ***p* < 0.01.


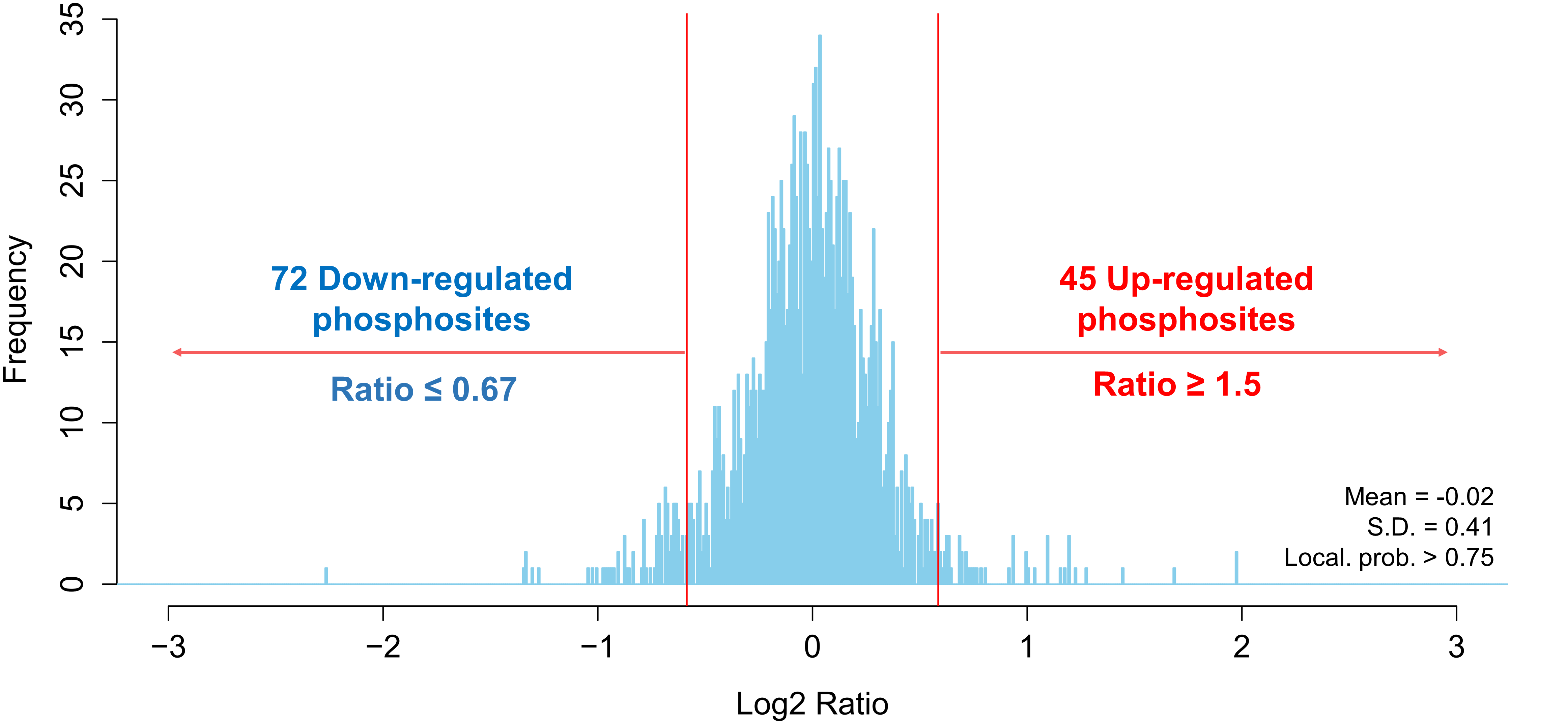


**Figure S2. Distribution of phosphorylated site ratios.**

The distribution of phosphosite H/L ratios in phosphoproteomic profile. The localization probability of phosphosites was filtered with greater than 0.75. A cutoff for significantly up- or down-regulated phosphosites were determined by 1.5 fold-change (ratio ≥ 1.5 or ≤ 0.67). 72 downregulated phosphosites and 45 upregulated phosphosites corresponding upon ZNF322A silenced A549 lung cancer cells.


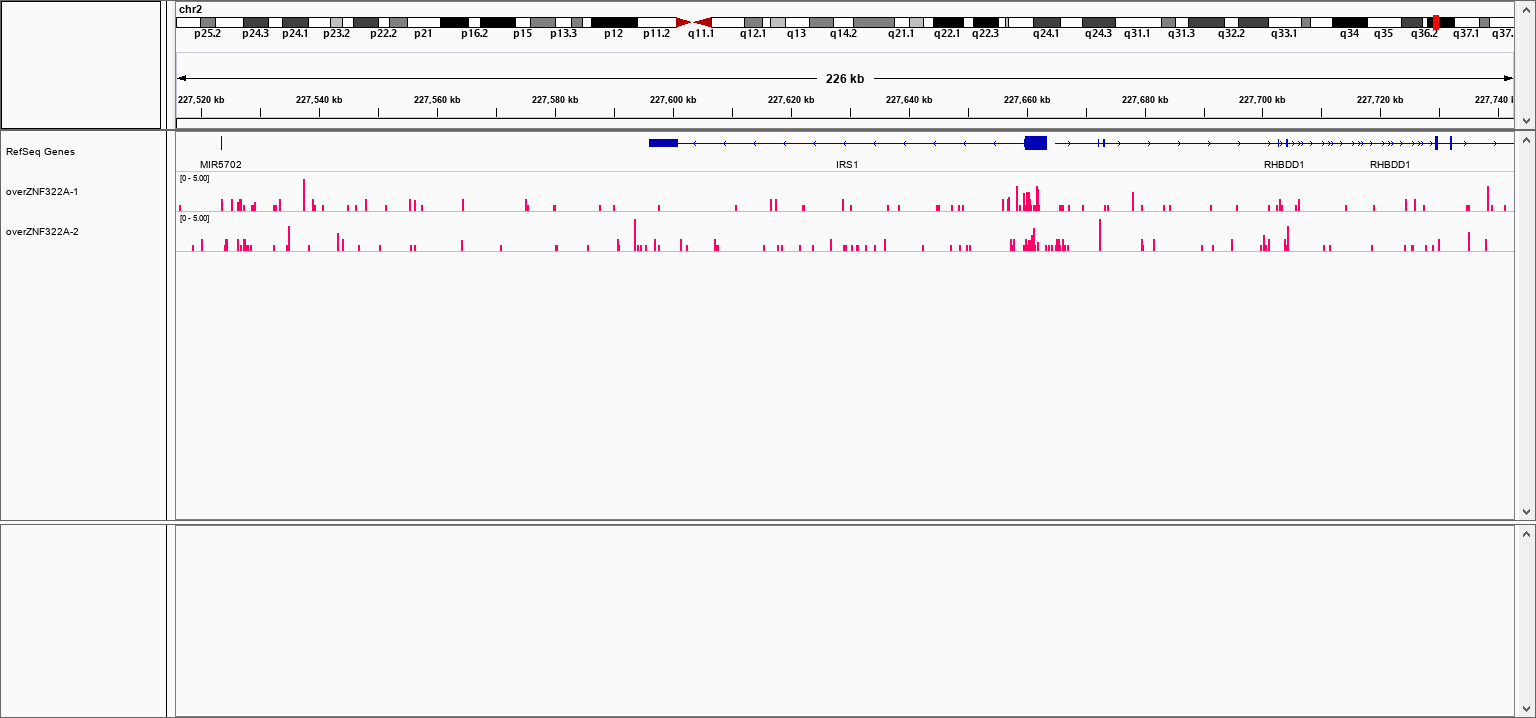


**Figure S3. Snapshot of the ChIP-seq binding profile of ZNF322A at IRS1 gene in lung cancer cell.**

ZNF322A binding sites were identified via chromatin immunoprecipitation-sequencing (ChIP-seq) analysis of HA-tagged ZNF322A overexpressing H460 lung cancer cells. ChIP-seq analyses were performed in duplicated samples using Applied Biosystems SOLiD system. The data is visulaized by Integrative Genomics Viewer (IGV) which was extracted from previous published anti-ZNF322A ChIP-seq profile. (https://www.ncbi.nlm.nih.gov; GSM2480405: overZNF322A-1 and GSM2480407: overZNF322A-2)


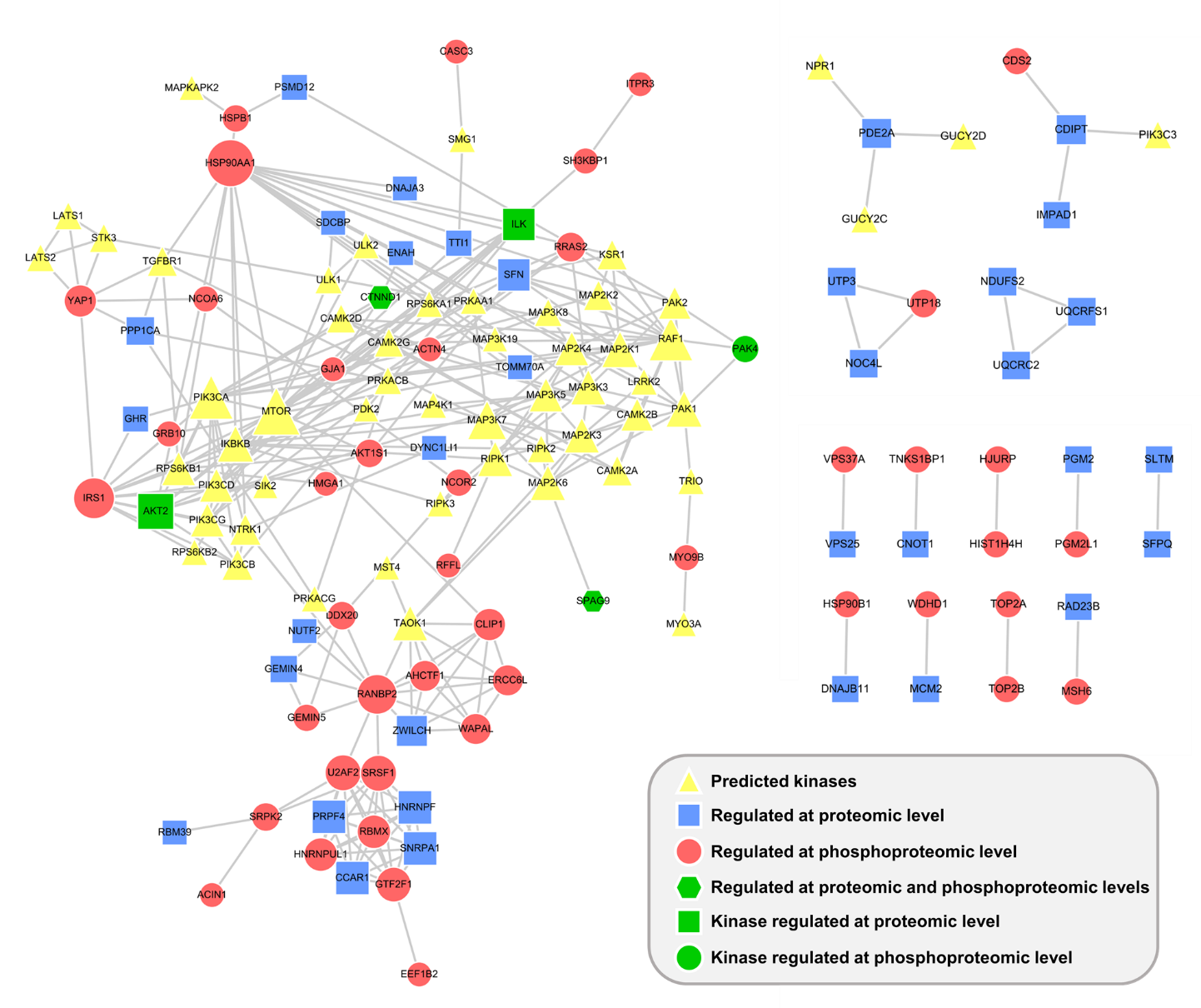


**Figure S4. Analysis of protein-phosphoprotein-kinase interactions in ZNF322A silenced A549 lung cancer cells.**

Differentially phosphorylated phosphosites sequence window were applied to kinase activity analysis using Dynapho. Kinases mapped to similar phosphorylated motif were grouped together. Protein-protein interaction of regulated proteins and predicted kinases were searched against STRING database and illustrated by Cytoscape.


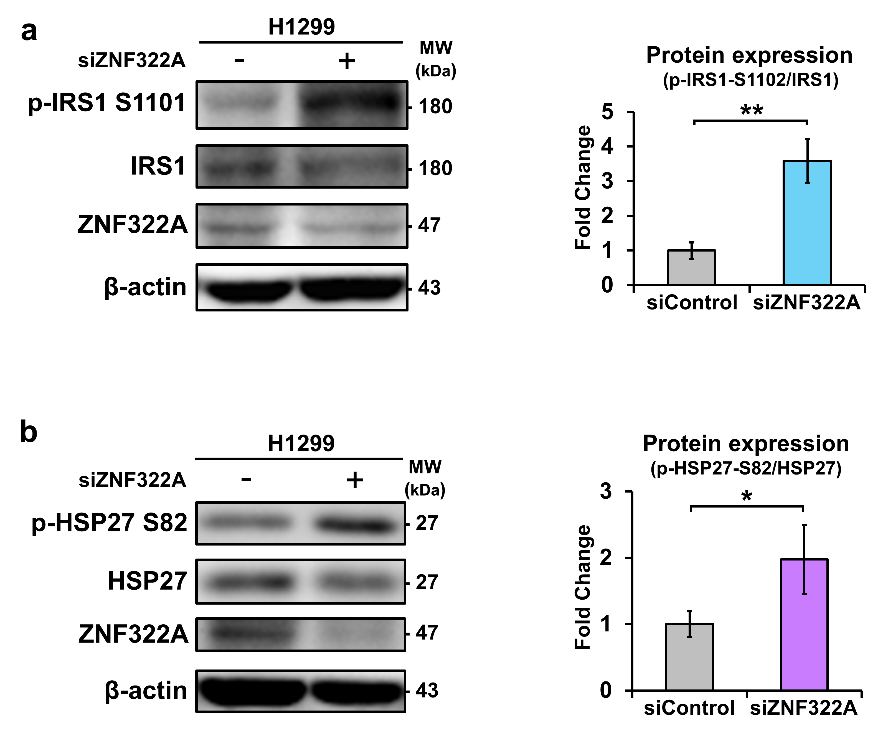


**Figure S5. IRS1^S1101^ and HSP27^S82^ phosphorylation** **in ZNF322A silenced H1299 lung cancer cells.** (a) Phosphorylation of IRS1^S1101^ and HSP27^S82^ were determined upon ZNF322A silenced H1299 lung cancer cells. Proteins were extracted from ZNF322A silenced H1299 lung cancer cells after 48 h transfection, and the protein expressions were examined by western blot analysis. Phosphorylation levels were determined and normalized to the level of total proteins. The normalized values were compared between siControl and siZNF322A groups. Actin was an internal control. **p* < 0.05; ***p* < 0.01.
