# Supplementary material for: ZNF322A-mediated protein phosphorylation induces autophagosome formation through modulation of IRS1-AKT glucose uptake and HSP-elicited UPR in lung cancer
Source: J Biomed Sci. 2020 Jun 23;27:75. doi: 10.1186/s12929-020-00668-5 (PMC7310457; doi:10.1186/s12929-020-00668-5)
Supplement: Supplementary file 2 — Additional file 2: Table S1. Sequence of primers and annealing conditions for real-time quantitative PCR. Table S2. The list of significantly regulated phosphosites of siZNF322A in A549 lung cancer cells. Table S3. Gene list of Gene Ontology analysis of siZNF322A in A549 lung cancer cells. Table S4. Signaling pathway enrichment analysis of siZNF322A phosphoproteomics in A549 lung cancer cells by REACTOME. [file 12929_2020_668_MOESM2_ESM.pdf]

Additional file 2: Supplementary Tables

ZNF322A-mediated protein phosphorylation induces autophagosome formation through modulation of IRS1-AKT glucose uptake and HSP-elicited UPR in lung cancer

Chantal Hoi Yin Cheung[1], Chia-Lang Hsu[1,2], Tsai-Yu Lin[1], Wei-Ting Chen[1], Yi-Ching Wang[3,4], Hsuan-Cheng Huang[5*], and Hsueh-Fen Juan[1,6*]

[1]Department of Life Science, National Taiwan University, Taipei 10617, Taiwan.
[2]Department of Medical Research, National Taiwan University Hospital, Taipei 10002, Taiwan
[3]Department of Pharmacology, College of Medicine, National Cheng Kung University, Tainan 70101, Taiwan.
[4]Institute of Basic Medical Sciences, College of Medicine, National Cheng Kung University, Tainan 70101, Taiwan.
[5]Institute of Biomedical Informatics, National Yang-Ming University, Taipei 11221, Taiwan.
[6]Graduate Institute of Biomedical Electronics and Bioinformatics, National Taiwan University, Taipei 10617, Taiwan.

Table S1. Sequence of primers and annealing conditions for real-time quantitative PCR.

Gene ID	Primer name	Sequence	Tm
79692	ZNF322A-Forward	5'-GTGGTCTGCGTGTGAGAGTGGC-3'	65.79
	ZNF322A-Reverse	5'- TTCTGACGCATGGGGAGGGCT-3'	65.44
415116	PIM3-Forward	5'- GGACAAGGAGAGCTTCGAGAAG-3'	60.42
	PIM3-Reverse	5'- CTCCTTCACCACGTGCTTCACA-3'	62.99
2597	GAPDH-Forward	5'-ACACCCACTCCTCCACCTTTG-3'	61.94
	GAPDH-Reverse	5'-GCTGTAGCCAAATTCGTTGTCATAC-3'	60.95

Table S2. The list of significantly regulated phosphosites of siZNF322A in A549 lung cancer cells.

No.[a]	Acc. No.[b]	Residue[c]	Position[d]	Description[e]	Gene name[f]	Phospho (STY) Prob.[g]	Local. Prob.[h]	*p*-value[i]	Score[j]	Phospho ratio[k]	Protein ratio[l]	Phospho /protein ratio[m]
**Up-regulated phosphosites**												
1	O15047	S	470	Histone-lysine N-methyltransferase SETD1A	SETD1A	S(0.149)GS(0.851)PAPETTNESVPFAQHSSIDSR	0.85	1.25E-12	99.60	2.01	N/A	N/A
2	O43707	S	159	Alpha-actinin-4	ACTN4	FAIQDIS(0.999)VEETSAK	1.00	8.24E-17	118.37	1.54	1.08	1.43
3	O60271	S	203	C-Jun-amino-terminal kinase-interacting protein 4	SPAG9	ERPIS(1)IGIFPIPAGDGIIT(1)PDAQK	1.00	6.18E-03	44.34	1.64	0.62	2.65
4	O60716	S	252	Catenin delta-1	CTNND1	APS(1)RQDVYGPQPQVR	1.00	7.51E-28	126.32	1.91	1.83	1.04
5	O60832	S	513	H/ACA ribonucleoprotein complex subunit 4	DKC1	EVEIVS(1)E	1.00	7.67E-07	94.11	1.62	N/A	N/A
6	O95292	S	156	Vesicle-associated membrane protein-associated protein B/C	VAPB	S(0.996)IS(0.102)S(0.245)S(0.592)IDDT(0.065)EVK	1.00	1.55E-03	61.65	1.60	N/A	N/A
7	P04792	S	82	Heat shock protein beta-1	HSPB1	QIS(1)SGVSEIR	1.00	1.22E-58	151.30	1.55	0.94	1.65
8	P07900	S	231	Heat shock protein HSP 90-alpha	HS90AA1	EVS(1)DDEAEEKEDKEEEK	1.00	1.53E-03	61.17	11.06	N/A	N/A
9	P17302	S	365	Gap junction alpha-1 protein	CJA1	IAAGHEIQPIAIVDQRPS(0.205)S(0.795)R	0.80	1.27E-03	47.50	1.62	N/A	N/A
10	P35568	S	1101	Insulin receptor substrate 1	IRS1	HS(0.111)S(0.882)ET(0.003)FS(0.001)S(0.001)TPSATR	0.88	5.26E-09	97.24	1.54	N/A	N/A
11	P35579	S	1943	Myosin-9	MYH9	GAGDGS(1)DEEVDGKADGAEAKPAE	1.00	5.03E-04	61.09	14.26	1.04	13.71

Table S2. continued

No.[a]	Acc. No.[b]	Residue[c]	Position[d]	Description[e]	Gene name[f]	Phospho (STY) Prob.[g]	Local. Prob.[h]	*p*-value[i]	Score[j]	Phospho ratio[k]	Protein ratio[l]	Phospho /protein ratio[m]
12	P51116	S	601	Fragile X mental retardation syndrome-related protein 2	FXR2	T(0.006)DGS(0.994)IS(1)GDRQPVTVADYISR	0.99	1.43E-31	125.37	1.73	N/A	N/A
13	Q02952	S	627	A-kinase anchor protein 12	AKAP12	RPS(1)ES(1)DKEDEIDK	1.00	1.86E-02	40.52	1.50	N/A	N/A
14	Q07955	S	199	Serine/arginine-rich splicing factor 1	SRSF1	VDGPRS(1)PS(0.55)Y(0.9)GRS(0.55)R	1.00	4.67E-03	62.53	2.29	0.90	2.54
15	Q09666	S	177	Neuroblast differentiation-associated protein AHNAK	AHNAK	DIDIS(0.012)S(0.988)PEFK	0.99	6.10E-35	136.93	1.67	N/A	N/A
16	Q09666	S	5841	Neuroblast differentiation-associated protein AHNAK	AHNAK	GHYEVTGS(1)DDETGK	1.00	2.73E-60	150.90	1.91	1.45	1.32
17	Q09666	S	5745	Neuroblast differentiation-associated protein AHNAK	AHNAK	S(0.913)S(0.587)KAS(0.587)IGS(0.913)IEGEAEAEAS(0.001)SPK	0.91	7.80E-05	72.77	2.25	1.45	1.55
18	Q13322	S	104	Growth factor receptor-bound protein 10	GRB10	SIQPQVS(1)PR	1.00	2.35E-04	77.53	1.53	N/A	N/A
19	Q13459	S	1290	Unconventional myosin-IXb	MYO9B	VQEKPDS(0.982)PGGS(0.015)T(0.003)QIQR	0.98	4.09E-04	69.26	1.57	N/A	N/A
20	Q14573	S	2670	Inositol 1,4,5-trisphosphate receptor type 3	ITPR3	IGFVDVQNCIS(1)R	1.00	5.69E-05	83.54	1.65	N/A	N/A
21	Q15149	S	4622	Plectin	PLEC	GYYSPYSVSGSGS(0.93)T(0.073)AGS(0.997)R	0.93	5.49E-82	176.70	1.89	1.12	1.69
22	Q5JSH3	S	403	WD repeat-containing protein 44	WDR44	EYVS(0.036)NDAAQS(0.964)DDEEK	0.96	7.18E-03	67.31	1.91	N/A	N/A
23	Q71RC2	S	722	La-related protein 4	LARP4	EQY(0.001)VPPRS(0.999)PK	1.00	4.04E-03	55.46	1.51	N/A	N/A
24	Q8IVL1	S	1480	Neuron navigator 2	NAV2	T(0.004)T(0.002)IS(0.018)ES(0.971)PIS(0.19)S(0.816)PAAS(1)PK	1.00	1.01E-05	88.33	2.14	N/A	N/A

Table S2. continued

No.[a]	Acc. No.[b]	Residue[c]	Position[d]	Description[e]	Gene name[f]	Phospho (STY) Prob.[g]	Local. Prob.[h]	*p*-value[i]	Score[j]	Phospho ratio[k]	Protein ratio[l]	Phospho /protein ratio[m]
25	Q8IZ41	S	377	Ras and EF-hand domain-containing protein	RASEF	SIHINNIS(0.958)PGNT(0.023)IS(0.018)R	0.96	1.16E-02	40.65	3.22	N/A	N/A
26	Q8NBN3	S	540	Transmembrane protein 87A	TMEM87A	WVEENVPSSVTDVAIPAIIDS(1)DEER	1.00	1.96E-17	105.61	2.34	N/A	N/A
27	Q8NEZ2	S	364	Vacuolar protein sorting-associated protein 37A	VPS37A	MEIDDFIS(1)S(1)FMEKR	1.00	1.09E-02	52.07	3.94	N/A	N/A
28	Q8WX93	S	893	Palladin	PALLD	IAS(1)DEEIQGTK	1.00	3.95E-05	87.52	1.68	N/A	N/A
29	Q8WZ73	S	240	E3 ubiquitin-protein ligase rififylin	RFFL	RAS(0.995)IS(0.005)DITDIEDIEGITVR	0.99	9.90E-05	73.52	1.50	N/A	N/A
30	Q96B97	S	587	SH3 domain-containing kinase-binding protein 1	SH3KBP1	ANS(0.977)PS(0.023)IFGTEGKPK	0.98	4.10E-03	53.95	1.70	1.39	1.22
31	Q96KC8	S	381	DnaJ homolog subfamily C member 1	DNAJC1	DS(0.022)VT(0.164)CS(0.813)PGMVR	0.81	2.46E-03	69.03	1.74	1.71	1.02
32	Q99543	S	60	DnaJ homolog subfamily C member 2	DNAJC2	EIS(1)EES(1)EDEEIQIEEFPMIK	1.00	7.82E-06	78.85	1.61	N/A	N/A
33	Q9BUJ2	S	194	Heterogeneous nuclear ribonucleoprotein U-like protein 1	HNRNPUL1	GRS(1)PQPPAEEDEDDFDDTIVAIDTYNCDIHFK	1.00	1.20E-06	74.39	1.54	N/A	N/A
34	Q9BXB4	S	178	Oxysterol-binding protein-related protein 11	OSBPL11	SFSIAS(0.003)S(0.812)S(0.124)NS(0.059)PIS(0.002)QR	0.81	4.22E-28	127.97	2.05	N/A	N/A
35	Q9C0C2	S	672	182 kDa tankyrase-1-binding protein	TNKS1BP1	TEAQDICRAS(1)PEPPGPESSSR	1.00	2.26E-05	81.64	1.54	1.38	1.12
36	Q9H2G2	S	779	STE20-like serine/threonine-protein kinase	SLK	DS(0.12)GS(0.88)ISIQETR	0.88	2.7E-07	93.42	2.72	N/A	N/A

Table S2. continued

No.[a]	Acc. No.[b]	Residue[c]	Position[d]	Description[e]	Gene name[f]	Phospho (STY) Prob.[g]	Local. Prob.[h]	*p*-value[i]	Score[j]	Phospho ratio[k]	Protein ratio[l]	Phospho /protein ratio[m]
37	Q9UHI6	S	677	Probable ATP-dependent RNA helicase DDX20	DDX20	S(0.554)Y(0.446)IEGS(1)S(1)DNQIK	1.00	2.6E-06	93.38	1.99	N/A	N/A
38	Q9UQ35	S	1014	Serine/arginine repetitive matrix protein 2	SRRM2	AQT(0.997)PPGPS(0.001)IS(0.003)GS(0.012)KS(0.988)PCPQEK	0.99	0.00097	60.86	2.42	1.37	1.77
39	Q9Y5J1	S	210	U3 small nucleolar RNA-associated protein 18 homolog	UTP18	T(0.002)S(0.074)S(0.924)DDES(1)EEDEDDIIQR	1.00	2E-161	229.68	2.28	N/A	N/A
40	Q9Y618	S	2288	Nuclear receptor corepressor 2	NCOR2	IT(0.011)ES(0.12)NS(0.872)AMVKS(0.997)K	1.00	0.00285	62.10	1.55	N/A	N/A
41	O60271	T	217	C-Jun-amino-terminal kinase-interacting protein 4	SPAG9	ERPIS(1)IGIFPIPAGDGIIT(1)PDAQK	1.00	0.00618	44.34	1.64	0.62	2.65
42	P42167	T	164	Lamina-associated polypeptide 2, isoforms beta/gamma	TMPO	S(0.006)S(0.006)T(0.006)PIPT(0.949)IS(0.029)S(0.003)S(0.001)AENTR	0.95	0.00011	76.31	1.52	0.70	2.17
43	Q09666	T	5845	Neuroblast differentiation-associated protein AHNAK	AHNAK	GHYEVT(0.018)GS(0.177)DDET(0.804)GK	0.80	0.00494	66.15	2.28	N/A	N/A
44	Q96B36	T	246	Proline-rich AKT1 substrate 1	AKT1S1	INT(0.992)S(0.008)DFQK	0.99	4.7E-09	99.34	1.69	1.24	1.36
45	O95218	Y	114	Zinc finger Ran-binding domain-containing protein 2	ZRANB2	ENVEY(0.95)IEREES(0.05)DGEYDEFGRK	0.95	3.7E-05	77.04	1.51	N/A	N/A
**Down-regulated phosphosites**												
1	A0FGR8	S	761	Extended synaptotagmin-2	ESYT2	EPT(0.151)PS(0.881)IAS(0.968)DIS(1)IPIATQEIR	1.00	6.5E-16	108.50	0.56	N/A	N/A

Table S2. continued

No.[a]	Acc. No.[b]	Residue[c]	Position[d]	Description[e]	Gene name[f]	Phospho (STY) Prob.[g]	Local. Prob.[h]	*p*-value[i]	Score[j]	Phospho ratio[k]	Protein ratio[l]	Phospho /protein ratio[m]
2	A3KN83	S	697	Protein strawberry notch homolog 1	SBNO1	IYSIIGIDITAPS(0.131)NNS(0.546)S(0.329)PRDS(0.994)PCK	0.99	1.3E-32	126.91	0.66	N/A	N/A
3	A8MT19	S	539	Putative rhophilin-2-like protein RHPN2P1	RHPN2P1	IS(0.004)FIS(0.964)WGT(0.032)NK	0.96	0.01216	45.28	0.54	N/A	N/A
4	O00264	S	181	Membrane-associated progesterone receptor component 1	PGRMC1	EGEEPTVYS(1)DEEEPK	1.00	3E-241	245.10	0.52	N/A	N/A
5	O00264	S	57	Membrane-associated progesterone receptor component 1	PGRMC1	GDQPAASGDS(1)DDDEPPPIPR	1.00	4E-198	231.08	0.61	N/A	N/A
6	O15061	S	429	Synemin	SYNM	T(0.001)FS(0.975)PT(0.013)Y(0.01)GIIR	0.98	0.0021	62.09	0.60	N/A	N/A
7	O15119	S	371	T-box transcription factor TBX3	TBX3	DICPS(1)EGES(1)DAEAESK	1.00	8.2E-10	91.89	0.64	N/A	N/A
8	O15234	S	363	Protein CASC3	CASC3	RIEQT(0.006)S(0.007)VRDPS(0.987)PEADAPVIGS(1)PEK	0.99	7.1E-06	78.04	0.64	N/A	N/A
9	O75717	S	868	WD repeat and HMG-box DNA-binding protein 1	WDHD1	NQVEEDAEDS(1)GEADDEEKPEIHK	1.00	4.7E-16	102.15	0.55	N/A	N/A
10	O95239	S	801	Chromosome-associated kinesin KIF4A	KIF4A	T(0.051)FS(0.839)IT(0.11)EVR	0.84	0.01032	54.49	0.51	N/A	N/A
11	O95674	S	21	Phosphatidate cytidylyltransferase 2	CDS2	VAHEPVAPPEDKES(0.826)ES(0.174)EAK	0.83	0.00034	84.37	0.60	N/A	N/A
12	O95810	S	293	Caveolae-associated protein 2	CAVIN2	VS(1)PITFGR	1.00	3.3E-13	104.22	0.62	N/A	N/A

Table S2. continued

No.[a]	Acc. No.[b]	Residue[c]	Position[d]	Description[e]	Gene name[f]	Phospho (STY) Prob.[g]	Local. Prob.[h]	*p*-value[i]	Score[j]	Phospho ratio[k]	Protein ratio[l]	Phospho /protein ratio[m]
13	O96013	S	181	Serine/threonine-protein kinase PAK 4	PAK4	RPIS(0.994)GPDVGT(0.006)PQPAGIASGAK	0.99	7.9E-07	81.32	0.48	N/A	N/A
14	P11388	S	1393	DNA topoisomerase 2-alpha	TOP2A	GS(0.001)VPIS(0.021)S(0.103)S(0.855)PPAT(0.021)HFPDETEITNPVPK	0.85	3.7E-12	100.14	0.51	0.87	0.59
15	P11388	S	1377	DNA topoisomerase 2-alpha	TOP2A	S(1)VVS(1)DIEADDVK	1.00	5E-122	197.47	0.65	0.87	0.75
16	P14625	S	306	Endoplasmin	HSP90B1	EES(1)DDEAAVEEEEEEK	1.00	0	288.30	0.58	1.07	0.54
17	P17096	S	44	High mobility group protein HMG-I/HMG-Y	HMGA1	KQPPVSPGTAIVGS(1)QK	1.00	0.00614	44.31	0.64	1.01	0.63
18	P18583	S	2029	Protein SON	SON	RFS(1)RS(1)PIR	1.00	0.01629	43.44	0.61	N/A	N/A
19	P18858	S	199	DNA ligase 1	LIG1	AET(0.999)PT(0.002)ES(0.995)VS(0.004)EPEVATK	1.00	2.9E-29	123.61	0.65	0.77	0.84
20	P24534	S	106	Elongation factor 1-beta	EEF1B2	DDDDIDIFGS(1)DDEEESEEAK	1.00	2E-249	245.11	0.60	1.11	0.54
21	P26368	S	79	Splicing factor U2AF 65 kDa subunit	U2AF2	EEHGGIIRS(1)PR	1.00	0.00136	72.10	0.59	1.07	0.55
22	P30622	S	195	CAP-Gly domain-containing linker protein 1	CLIP1	T(0.001)AS(0.005)ES(0.994)IS(0.003)NIS(0.998)EAGS(1)IK	0.99	4E-38	135.63	0.64	N/A	N/A
23	P30622	S	204	CAP-Gly domain-containing linker protein 1	CLIP1	T(0.001)AS(0.005)ES(0.994)IS(0.003)NIS(0.998)EAGS(1)IK	1.00	5E-128	201.29	0.65	N/A	N/A
24	P38159	S	352	RNA-binding motif protein, X chromosome	RBMX	GIPPS(1)MERGY(1)PPPR	1.00	0.01063	42.31	0.67	0.96	0.70
25	P46013	S	584	Proliferation marker protien KI-67	MKI67	AQSIVIS(0.013)PPAPS(0.987)PR	0.99	0.00708	63.30	0.64	1.01	0.63

Table S2. continued

No.[a]	Acc. No.[b]	Residue[c]	Position[d]	Description[e]	Gene name[f]	Phospho (STY) Prob.[g]	Local. Prob.[h]	*p*-value[i]	Score[j]	Phospho ratio[k]	Protein ratio[l]	Phospho /protein ratio[m]
26	P49792	S	2297	E3 SUMO-protein ligase RanBP2	RANBP2	INQS(0.364)GT(0.364)S(0.266)VGT(0.225)DEES(0.777)DVT(0.005)QEEER	0.78	8.6E-05	68.95	0.63	N/A	N/A
27	P50502	S	79	Hsc70-interacting protein	ST13	KVEEDIKADEPS(1)S(1)EES(1)DIEIDK	1.00	9E-126	235.21	0.64	1.12	0.57
28	P52701	S	252	DNA mismatch repair protein Msh6	MSH6	VIS(1)DS(1)ES(1)DIGGS(1)DVEFKPDTK	1.00	5E-08	89.30	0.62	1.10	0.56
29	P55209	S	10	Nucleosome assembly protein 1-like 1	NAP1L1	EQS(1)EIDQDIDDVEEVEEEETGEETK	1.00	4.1E-33	121.56	0.55	1.28	0.43
30	P62070	S	186	Ras-related protein R-Ras2	RRAS2	FQEQECPPS(0.996)PEPT(0.004)RK	1.00	0.01941	49.15	0.52	N/A	N/A
31	P62805	S	48	Histone H4	H4C1	RIS(1)GIIYEETR	1.00	0.00098	70.94	0.65	N/A	N/A
32	P78362	S	380	SRSF protein kinase 2	SRPK2	DEDDVDQEIANIDPT(0.002)WIES(0.998)PK	1.00	3.5E-16	106.70	0.55	N/A	N/A
33	Q02880	S	1400	DNA topoisomerase 2-beta	TOP2B	AS(0.86)PIT(0.14)NDGEDEFVPS(1)DGIDK	1.00	4.7E-23	116.19	0.40	1.12	0.36
34	Q14157	S	608	Ubiquitin-associated protein 2-like	UBAP2L	RY(0.063)PS(0.351)S(0.44)IS(0.152)S(0.831)S(0.163)PQK	0.83	0.00053	71.09	0.60	1.18	0.51
35	Q14686	S	114	Nuclear receptor coactivator 6	NCOA6	IIAQS(1)NNQQIR	1.00	0.01502	41.69	0.02	N/A	N/A
36	Q2NKX8	S	1134	DNA excision repair protein ERCC-6-like	ERCC6L	GPEDYPEEGVEES(1)S(0.998)GEAS(0.002)K	1.00	1.9E-10	94.01	0.66	N/A	N/A
37	Q5T4S7	S	2718	E3 ubiquitin-protein ligase UBR4	UBR4	RRHVT(1)IPS(1)S(1)PR	1.00	0.001	69.72	0.65	N/A	N/A

Table S2. continued

No.[a]	Acc. No.[b]	Residue[c]	Position[d]	Description[e]	Gene name[f]	Phospho (STY) Prob.[g]	Local. Prob.[h]	*p*-value[i]	Score[j]	Phospho ratio[k]	Protein ratio[l]	Phospho /protein ratio[m]
38	Q66K74	S	655	Microtubule-associated protein 1S	MAP1S	S(0.001)PAEGS(0.004)ERIS(0.996)IS(1)PIR	1.00	0.00917	49.15	0.65	N/A	N/A
39	Q68CZ2	S	660	Tensin-3	TNS3	GVGSGPHPPDT(0.004)QQPS(0.937)PS(0.059)K	0.94	3.1E-05	77.19	0.49	N/A	N/A
40	Q68CZ2	S	662	Tensin-3	TNS3	GVGSGPHPPDTQQPS(0.204)PS(0.796)K	0.80	0.00038	64.84	0.50	N/A	N/A
41	Q6PCE3	S	175	Glucose 1,6-bisphosphate synthase	PGM2L1	AVAGVMIT(0.059)AS(0.941)HNR	0.94	0.00344	51.00	0.39	N/A	N/A
42	Q7Z5K2	S	221	Wings apart-like protein homolog	WAPL	RPES(0.839)PS(0.162)EIS(0.999)PIK	1.00	0.01951	49.63	0.67	N/A	N/A
43	Q8NCD3	S	473	Holliday junction recognition protein	HJURP	GGPAS(1)PGGIQGIETR	1.00	0.00253	58.84	0.56	N/A	N/A
44	Q8TDM6	S	1263	Disks large homolog 5	DLG5	IGS(0.954)S(0.023)S(0.023)NIQFK	0.95	0.00578	50.09	0.63	N/A	N/A
45	Q8TEQ6	S	778	Gem-associated protein 5	GEMIN5	ENSGPVENGVS(1)DQEGEEQAR	1.00	8.3E-23	120.82	0.41	0.97	0.42
46	Q8WYP5	S	1283	Protein ELYS	AHCTF1	TTS(0.001)FFINS(0.999)PEK	1.00	0.00185	61.65	0.64	N/A	N/A
47	Q96C19	S	76	EF-hand domain-containing protein D2	EFHD2	RADINQGIGEPQS(0.212)PS(0.788)R	0.79	2.2E-15	105.63	0.40	1.05	0.38
48	Q96CP6	S	263	GRAM domain-containing protein 1A	GRAMD1A	EVGDVIAIS(0.004)DIT(0.002)S(0.003)S(0.002)GAADRS(0.98)QEPS(0.718)PVGS(0.291)R	0.98	4.6E-09	86.08	0.62	N/A	N/A
49	Q9HCG1	S	715	Zinc finger protein 160	ZNF160	AFS(0.994)VRS(0.478)S(0.478)IT(0.247)T(0.803)HQAIHTGKK	0.99	0.00453	55.01	0.64	N/A	N/A

Table S2. continued

No.[a]	Acc. No.[b]	Residue[c]	Position[d]	Description[e]	Gene name[f]	Phospho (STY) Prob.[g]	Local. Prob.[h]	*p*-value[i]	Score[j]	Phospho ratio[k]	Protein ratio[l]	Phospho /protein ratio[m]
50	Q9BX95	S	112	Sphingosine-1-phosphate phosphatase 1	SGPP1	RNS(1)ITGEEGQIAR	1.00	8.6E-90	180.76	0.53	N/A	N/A
51	Q9BXP5	S	67	Serrate RNA effector molecule homolog	SRRT	ERFS(1)PPRHEIS(1)PPQK	1.00	0.00443	55.71	0.61	N/A	N/A
52	Q9H6F5	S	102	Coiled-coil domain-containing protein 86	CCDC86	QQDIHIES(1)PQRQPEY(0.152) S(0.835)PES(0.013)PR	1.00	1.7E-31	121.26	0.65	N/A	N/A
53	Q9H6Z4	S	108	Ran-binding protein 3	RANBP3	SAGGSSPEGGEDS(1)DREDGN YCPPVK	1.00	1.1E-11	92.93	0.65	N/A	N/A
54	Q9HB09	S	242	Bcl-2-like protein 12	BCL2L12	IVRIS(0.961)S(0.036)DS(0.003)F AR	0.96	0.01064	45.28	0.61	N/A	N/A
55	Q9HCK8	S	2069	Chromodomain-helicase-DNA-binding protein 8	CHD8	IEDEDDS(1)DS(1)EIDISK	1.00	0.00165	102.24	0.63	N/A	N/A
56	Q9NRQ2	S	257	Phospholipid scramblase 4	PLSCR4	VRGPCSTYGCGS(0.776)DS(0.2 23)VFEVK	0.78	0.00096	57.94	0.04	N/A	N/A
57	Q9P1Y6	S	915	PHD and RING finger domain-containing protein 1	PHRF1	GAVAAEGAS(0.876)DT(0.124) EREEPTESQGIAAR	0.88	2E-56	147.49	0.61	N/A	N/A
58	Q9UKV3	S	240	Apoptotic chromatin condensation inducer in the nucleus	ACIN1	IS(1)EGS(1)QPAEEEEDQETPS R	1.00	3.7E-07	87.20	0.58	N/A	N/A
59	Q9ULL5	S	560	Proline-rich protein 12	PRR12	NIETIPS(0.071)FS(0.843)S(0.926)DEEDS(0.161)VAK	0.93	5.8E-32	128.51	0.63	N/A	N/A
60	Q9UPN3	S	3929	Microtubule-actin cross-linking factor 1, isoforms 1/2/3/5	MACF1	RQGS(0.04)FS(0.96)EDVISHK	0.96	2.2E-07	89.48	0.63	1.03	0.61

Table S2. continued

No.[a]	Acc. No.[b]	Residue[c]	Position[d]	Description[e]	Gene name[f]	Phospho (STY) Prob.[g]	Local. Prob.[h]	*p*-value[i]	Score[j]	Phospho ratio[k]	Protein ratio[l]	Phospho /protein ratio[m]
61	Q9UQ35	S	1320	Serine/arginine repetitive matrix protein 2	SRRM2	EIS(1)NS(1)PIRENS(0.07)FGS(0.93)PIEFR	1.00	9.8E-42	133.28	0.58	1.37	0.42
62	Q9UQ35	S	2020	Serine/arginine repetitive matrix protein 2	SRRM2	S(1)RT(1)PPAIR	1.00	0.0075	62.47	0.52	1.37	0.38
63	Q9UQ35	S	295	Serine/arginine repetitive matrix protein 2	SRRM2	T(0.004)HT(0.057)T(0.057)AIAGRS(0.901)PS(0.969)PAS(0.012)GR	0.90	0.01127	54.31	0.58	1.37	0.42
64	Q9Y5S2	S	1690	Serine/threonine-protein kinase MRCK beta	CDC42BPB	HSTPSNSSNPSGPPS(0.871)PNS(0.128)PHR	0.87	0.00114	51.69	0.21	N/A	N/A
65	P11388	T	1397	DNA topoisomerase 2-alpha	TOP2A	GSVPIS(0.004)S(0.038)S(0.144)PPAT(0.786)HFPDET(0.015)EIT(0.013)NPVPK	0.79	6.7E-05	75.23	0.53	0.87	0.61
66	P11388	T	1244	DNA topoisomerase 2-alpha	TOP2A	NENT(0.955)EGS(0.045)PQEDGVEIEGIK	0.96	2.4E-42	137.45	0.62	0.87	0.71
67	P35269	T	389	General transcription factor IIF subunit 1	GTF2F1	GNS(1)RPGT(0.998)PS(0.002)AEGGSTSSTIR	1.00	2.1E-06	87.55	0.67	N/A	N/A
68	P78362	T	498	SRSF protein kinase 2	SRPK2	TVS(0.492)AS(0.323)S(0.396)T(0.788)GDIPK	0.79	0.00228	81.71	0.54	N/A	N/A
69	Q14980	T	2000	Nuclear mitotic apparatus protein 1	NUMA1	VSIEPHQGPGT(0.998)PES(0.002)K	1.00	2.7E-06	84.76	0.53	1.08	0.49
70	Q8N3X1	T	479	Formin-binding protein 4	FNBP4	T(0.057)GRDT(0.936)PENGET(0.007)AIGAENSEK	0.94	0.00204	65.02	0.58	N/A	N/A
71	Q9HCG1	T	722	Zinc finger protein 160	ZNF160	AFS(0.994)VRS(0.478)S(0.478)IT(0.247)T(0.803)HQAIHTGKK	0.80	0.00453	55.01	0.64	N/A	N/A

Table S2. continued

No.[a]	Acc. No.[b]	Residue[c]	Position[d]	Description[e]	Gene name[f]	Phospho (STY) Prob.[g]	Local. Prob.[h]	*p*-value[i]	Score[j]	Phospho ratio[k]	Protein ratio[l]	Phospho /protein ratio[m]
72	P38159	Y	357	RNA-binding motif protein, X chromosome	RBMX	GIPPS(1)MERGY(1)PPPR	1.00	0.01063	42.31	0.67	0.96	0.70

[a]Identifier of this table. [b]Unique identifier for proteins in Uniprot database. [c]The residue of phosphorylation within this peptide. [d]The position of the phosphorylated residues within the leading protein of this peptide. [e]The name of identified proteins this peptide is associated with. [f]The name of identified genes this peptide is associated with. [g]The sequence of matched peptide including the location(s) of phosphorylated amino acid. [h]The localization probability of phosphorylated residue at the position[d] of this protein. [i]The posterior error probability of the best identified modified peptide containing this site. [j]The score for the best associated MS/MS spectrum in Andromenda. The higher the better. [k]Normalized ratio of siZNF322A/Control ratio from phosphoproteome. [l]Normalized ratio of the raw siZNF322A/Control ratio from phoproteome. [m]Normalized ratio of phosphoprotein to its protein.

Table S3. Gene list of Gene Ontology analysis of siZNF322A in A549 lung cancer cells.

GOID	Description	P-value	Hits	Gene List	Number of genes	
					proteomics	Phosphor-proteomics
GO:0016071	mRNA metabolic process	3.37E-17	24/844	RBMX|DNAJB11|U2AF2|GEMIN5|HNRNPUL1|TNKS1BP1|GTF2F1|HSPB1|PSMD12|ZRANB2|SON|SNRPA1|CCAR1|PHRF1|SFPQ|HNRNPF|EDC4|PRPF4|ACIN1|CASC3|SRRM2|CNOT1|SRSF1|RBM39	10	14
GO:0010467	gene expression	6.19E-16	21/677	SNRPA1|GTF2F1|HNRNPUL1|NCOR2|RBMX|HNRNPF|TNKS1BP1|CCAR1|PRPF4|HSPB1|U2AF2|SRSF1|GEMIN4|GEMIN5|DDX20|PSMD12|CNOT1|CASC3|YAP1|EEF1B2|EDC4	8	13
GO:0006397	mRNA processing	9.11E-16	20/610	SNRPA1|GTF2F1|PHRF1|SRRM2|SFPQ|ACIN1|TNKS1BP1|PRPF4|RBM39|U2AF2|SRSF1|HNRNPF|GEMIN5|CCAR1|RBMX|HNRNPUL1|CNOT1|CASC3|SON|ZRANB2	7	13
GO:0008380	RNA splicing	9.27E-16	18/449	SNRPA1|GTF2F1|HNRNPUL1|SRRM2|SFPQ|ACIN1|PRPF4|RBM39|U2AF2|SRSF1|HNRNPF|GEMIN5|CCAR1|RBMX|CASC3|SON|ZRANB2|SRPK2	6	12
GO:0034655	nucleobase-containing compound catabolic process	1.21E-13	21/926	CHD8|TOP2B|ATP5PB|TNKS1BP1|EDC4|PDE2A|HMGA1|MAP1S|TOP2A|ACIN1|KIF4A|RAD23B|DDX20|MSH6|HSP90AA1|CNOT1|MACF1|CASC3|HMGB2|RAB21|MYH9	7	14
GO:0000398	mRNA splicing, via spliceosome	1.81E-11	12/252	SNRPA1|GTF2F1|HNRNPUL1|SRRM2|SFPQ|HNRNPF|PRPF4|U2AF2|SRSF1|GEMIN5|CCAR1|RBMX	5	7
GO:0000377	RNA splicing, via transesterification reactions with bulged adenosine as nucleophile	1.81E-11	12/252	SNRPA1|GTF2F1|HNRNPUL1|SRRM2|SFPQ|HNRNPF|PRPF4|U2AF2|SRSF1|GEMIN5|CCAR1|RBMX	5	7
GO:0000375	RNA splicing, via transesterification reactions	2.29E-11	12/260	SNRPA1|GTF2F1|HNRNPUL1|SRRM2|SFPQ|HNRNPF|PRPF4|U2AF2|SRSF1|GEMIN5|CCAR1|RBMX	5	7

Table S3. continued

GOID	Description	P-value	Hits	Gene List	Number of genes	
					proteomics	Phosphor-proteomics
GO: 0006200	ATP catabolic process	3.24E-11	11/203	ATP5PB|TOP2A|ACIN1|KIF4A|DDX20|MACF1|HSP90AA1|TOP2B|MSH6|CHD8|MYH9	1	10
GO:0009158	ribonucleoside monophosphate catabolic process	3.52E-11	11/209	ATP5PB|TOP2A|ACIN1|KIF4A|DDX20|MACF1|HSP90AA1|TOP2B|MSH6|CHD8|MYH9	1	10
GO:0009169	purine ribonucleoside monophosphate catabolic process	3.52E-11	11/209	ATP5PB|TOP2A|ACIN1|KIF4A|DDX20|MACF1|HSP90AA1|TOP2B|MSH6|CHD8|MYH9	1	10
GO:0009128	purine nucleoside monophosphate catabolic process	3.52E-11	11/210	ATP5PB|TOP2A|ACIN1|KIF4A|DDX20|MACF1|HSP90AA1|TOP2B|MSH6|CHD8|MYH9	1	10
GO:0009125	nucleoside monophosphate catabolic process	3.79E-11	11/213	ATP5PB|TOP2A|ACIN1|KIF4A|DDX20|MACF1|HSP90AA1|TOP2B|MSH6|CHD8|MYH9	1	10
GO:0000278	mitotic cell cycle	4.36E-10	13/444	MCM2|H4C9|H4C1|H4C4|H4C6|H4C12|H4C11|H4C3|H4C8|H4C2|H4C5|H4C13|H4C14|H4C16|H4C15|ERCC6L|RANBP2|CLIP1|AHCTF1|TOP2A|WAPL|HSP90AA1|LIG1|NUMA1|ZWILCH|PSMD12	3	10
GO:0007049	cell cycle	7.91E-10	17/950	RANBP2|CCAR1|MCM2|H4C9|H4C1|H4C4|H4C6|H4C12|H4C11|H4C3|H4C8|H4C2|H4C5|H4C13|H4C14|H4C16|H4C15|ERCC6L|PPP1CA|CLIP1|AHCTF1|TOP2A|WAPL|HSP90AA1|LIG1|PSMD12|PAK4|NUMA1|ZWILCH|HJURP	5	12
GO:0009154	purine ribonucleotide catabolic process	1.20E-09	13/489	TOP2B|ATP5PB|TOP2A|ACIN1|KIF4A|DDX20|MACF1|HSP90AA1|PDE2A|MSH6|CHD8|RAB21|MYH9	3	10
GO:0009261	ribonucleotide catabolic process	1.20E-09	13/490	TOP2B|ATP5PB|TOP2A|ACIN1|KIF4A|DDX20|MACF1|HSP90AA1|PDE2A|MSH6|CHD8|RAB21|MYH9	3	10
GO:0006195	purine nucleotide catabolic process	1.68E-09	13/506	TOP2B|ATP5PB|TOP2A|ACIN1|KIF4A|DDX20|MACF1|HSP90AA1|PDE2A|MSH6|CHD8|RAB21|MYH9	3	10

Table S3. continued

GOID	Description	P-value	Hits	Gene List	Number of genes proteomics	Number of genes Phosphor-proteomics
GO:0072523	purine-containing compound catabolic process	2.12E-09	13/518	TOP2B|ATP5PB|TOP2A|ACIN1|KIF4A|DDX20|MACF1|HSP90AA1|PDE2A|MSH6|CHD8|RAB21|MYH9	3	10
GO:0043484	regulation of RNA splicing	3.09E-09	8/119	AHNAK|RBMX|HNRNPF|SRSF1|U2AF2|ACIN1|SON|SRPK2	1	7
GO:0046434	organophosphate catabolic process	3.36E-09	14/660	PGM2|TOP2B|ATP5PB|TOP2A|ACIN1|KIF4A|DDX20|MACF1|HSP90AA1|PDE2A|MSH6|CHD8|RAB21|MYH9	4	10
GO:0009203	ribonucleoside triphosphate catabolic process	4.36E-09	12/449	ATP5PB|TOP2A|ACIN1|KIF4A|DDX20|MACF1|HSP90AA1|TOP2B|MSH6|CHD8|RAB21|MYH9	2	10
GO:0009207	purine ribonucleoside triphosphate catabolic process	4.36E-09	12/449	ATP5PB|TOP2A|ACIN1|KIF4A|DDX20|MACF1|HSP90AA1|TOP2B|MSH6|CHD8|RAB21|MYH9	2	10
GO:0046130	purine ribonucleoside catabolic process	4.82E-09	12/461	ATP5PB|TOP2A|ACIN1|KIF4A|DDX20|MACF1|HSP90AA1|TOP2B|MSH6|CHD8|RAB21|MYH9	2	10
GO:0009143	nucleoside triphosphate catabolic process	4.82E-09	12/459	ATP5PB|TOP2A|ACIN1|KIF4A|DDX20|MACF1|HSP90AA1|TOP2B|MSH6|CHD8|RAB21|MYH9	2	10
GO:0009146	purine nucleoside triphosphate catabolic process	4.82E-09	12/455	ATP5PB|TOP2A|ACIN1|KIF4A|DDX20|MACF1|HSP90AA1|TOP2B|MSH6|CHD8|RAB21|MYH9	2	10
GO:0009166	nucleotide catabolic process	4.82E-09	13/569	TOP2B|ATP5PB|TOP2A|ACIN1|KIF4A|DDX20|MACF1|HSP90AA1|PDE2A|MSH6|CHD8|RAB21|MYH9	3	10
GO:0006152	purine nucleoside catabolic process	4.82E-09	12/461	ATP5PB|TOP2A|ACIN1|KIF4A|DDX20|MACF1|HSP90AA1|TOP2B|MSH6|CHD8|RAB21|MYH9	2	10
GO:1901292	nucleoside phosphate catabolic process	5.12E-09	13/577	TOP2B|ATP5PB|TOP2A|ACIN1|KIF4A|DDX20|MACF1|HSP90AA1|PDE2A|MSH6|CHD8|RAB21|MYH9	3	10
GO:0042454	ribonucleoside catabolic process	5.59E-09	12/470	ATP5PB|TOP2A|ACIN1|KIF4A|DDX20|MACF1|HSP90AA1|TOP2B|MSH6|CHD8|RAB21|MYH9	2	10

Table S3. continued

GOID	Description	P-value	Hits	Gene List	Number of genes	
					proteomics	Phosphor-proteomics
GO:1901136	carbohydrate derivative catabolic process	6.82E-09	14/719	PGM2|TOP2B|ATP5PB|TOP2A|ACIN1|KIF4A|DDX20|MACF1|HSP90AA1|PDE2A|MSH6|CHD8|RAB21|MYH9	4	10
GO:0009164	nucleoside catabolic process	6.96E-09	12/482	ATP5PB|TOP2A|ACIN1|KIF4A|DDX20|MACF1|HSP90AA1|TOP2B|MSH6|CHD8|RAB21|MYH9	2	10
GO:1901658	glycosyl compound catabolic process	7.93E-09	12/489	ATP5PB|TOP2A|ACIN1|KIF4A|DDX20|MACF1|HSP90AA1|TOP2B|MSH6|CHD8|RAB21|MYH9	2	10
GO:0061024	membrane organization	1.03E-08	15/886	AP1G1|ZMPSTE24|SFN|GJA1|DNAJA3|RANBP2|GNPAT|MYO18A|VPS37A|SNX3|HSP90AA1|ATP1B3|VPS25|PLSCR4|AKT2	10	5
GO:0006325	chromatin organization	1.15E-08	15/895	UTP3|DNMT1|H4C9|H4C1|H4C4|H4C6|H4C12|H4C11|H4C3|H4C8|H4C2|H4C5|H4C13|H4C14|H4C16|H4C15|HUWE1|SETD1A|SFPQ|HMGA1|WDHD1|HMGB2|DNAJC2|HMGN1|HJURP|NAP1L1|MCM2|CHD8	7	8
GO:0044403	symbiosis, encompassing mutualism through parasitism	5.15E-07	12/723	UBR4|AP1G1|VPS37A|HMGA1|GTF2F1|RANBP2|WAPL|VAPB|DYNC1LI1|CCDC86|VDAC1|PSMD12	4	8
GO:0016032	viral process	5.15E-07	12/723	UBR4|AP1G1|VPS37A|HMGA1|GTF2F1|RANBP2|WAPL|VAPB|DYNC1LI1|CCDC86|VDAC1|PSMD12	4	8
GO:0044764	multi-organism cellular process	5.40E-07	12/728	UBR4|AP1G1|VPS37A|HMGA1|GTF2F1|RANBP2|WAPL|VAPB|DYNC1LI1|CCDC86|VDAC1|PSMD12	4	8
GO:0050684	regulation of mRNA processing	5.74E-07	6/101	RBMX|CNOT1|U2AF2|ACIN1|SRSF1|SRPK2	1	5
GO:0032508	DNA duplex unwinding	1.75E-06	5/65	MCM2|HMGA1|TOP2B|TOP2A|CHD8	1	4
GO:0044419	interspecies interaction between organisms	1.75E-06	12/818	UBR4|AP1G1|VPS37A|HMGA1|GTF2F1|RANBP2|WAPL|VAPB|DYNC1LI1|CCDC86|VDAC1|PSMD12	4	8
GO:0048024	regulation of mRNA splicing, via spliceosome	1.85E-06	5/66	ACIN1|RBMX|SRPK2|U2AF2|SRSF1	0	5

Table S3. continued

GOID	Description	P-value	Hits	Gene List	Number of genes	
					proteomics	Phosphor-proteomics
GO:0032392	DNA geometric change	1.90E-06	5/67	MCM2|HMGA1|TOP2B|TOP2A|CHD8	1	4
GO:0032075	positive regulation of nuclease activity	1.90E-06	5/67	HMGB2|VAPB|DNAJB11|ADD1|HSP90B1	3	2
GO:0051345	positive regulation of hydrolase activity	2.13E-06	12/842	DNAJA3|ITPR3|DNAJB11|HMGB2|DNAJC10|DNAJC1|MSH6|HSP90B1|ADD1|ATP1B3|VAPB|AKT2	7	5
GO:0032069	regulation of nuclease activity	2.80E-06	5/73	HMGB2|VAPB|DNAJB11|ADD1|HSP90B1	3	2
GO:0071103	DNA conformation change	3.00E-06	7/221	MCM2|HMGA1|TOP2A|HMGB2|TOP2B|ACIN1|CHD8	2	5
GO:0006457	protein folding	5.37E-06	10/611	DNAJA3|RANBP2|DNAJB11|DNAJC10|DNAJC1|DNAJC2|ST13|HSP90AA1|VBP1|HSP90B1	4	6
GO:0006986	response to unfolded protein	8.48E-06	6/166	HSPB1|DNAJB11|VAPB|HSP90B1|ADD1|HSP90AA1	2	4
GO:0016568	chromatin modification	1.45E-05	10/687	UTP3|DNMT1|H4C9|H4C1|H4C4|H4C6|H4C12|H4C11|H4C3|H4C8|H4C2|H4C5|H4C13|H4C14|H4C16|H4C15|HUWE1|SETD1A|SFPQ|HMGA1|DNAJC2|HJURP|CHD8	4	6
GO:0035966	response to topologically incorrect protein	1.48E-05	6/184	HSPB1|DNAJB11|VAPB|HSP90B1|ADD1|HSP90AA1	2	4
GO:0022411	cellular component disassembly	1.83E-05	8/417	PRSS1|HMGA1|RANBP2|HMGB2|PLEC|VIL1|ACIN1|ADD1	4	4
GO:0034329	cell junction assembly	1.87E-05	7/297	GJA1|GNPAT|DLG5|PLEC|ACTN4|ILK|CTNND1	3	5
GO:0034728	nucleosome organization	4.17E-05	6/223	MCM2|H4C9|H4C1|H4C4|H4C6|H4C12|H4C11|H4C3|H4C8|H4C2|H4C5|H4C13|H4C14|H4C16|H4C15|HMGA1|HMGB2|HJURP|NAP1L1	2	4
GO:0034330	cell junction organization	7.19E-05	7/368	GJA1|GNPAT|DLG5|PLEC|ACTN4|ILK|CTNND1	3	5
GO:0015931	nucleobase-containing compound transport	7.32E-05	6/248	GJA1|AHCTF1|RANBP2|U2AF2|SRSF1|CASC3	0	6
GO:0048285	organelle fission	7.61E-05	8/515	ERCC6L|CLIP1|WAPL|DYNC1LI1|MTFR2|MKI67|NUMA1|ZWILCH	3	5

Table S3. continued

GOID	Description	P-value	Hits	Gene List	Number of genes proteomics	Number of genes Phosphor-proteomics
GO:0032269	negative regulation of cellular protein metabolic process	8.70E-05	10/861	AKT1S1|DNMT1|SFN|DNAJA3|HSPB1|DNAJC10|DNAJC1|ST13|PSMD12|ILK	6	4
GO:0006310	DNA recombination	9.23E-05	7/387	NCOA6|SFPQ|TOP2A|HMGB2|MSH6|LIG1|TOP2B	2	5
GO:0044802	single-organism membrane organization	9.27E-05	9/695	ZMPSTE24|DNAJA3|RANBP2|MYO18A|SNX3|HSP90AA1|ATP1B3|PLSCR4|AKT2	6	3
GO:0071824	protein-DNA complex subunit organization	9.93E-05	6/266	MCM2|H4C9|H4C1|H4C4|H4C6|H4C12|H4C11|H4C3|H4C8|H4C2|H4C5|H4C13|H4C14|H4C16|H4C15|HMGA1|HMGB2|HJURP|NAP1L1	2	4
GO:0022618	ribonucleoprotein complex assembly	1.15E-04	6/274	LUC7L|SRSF1|GEMIN4|GEMIN5|DDX20|SRPK2	2	4
GO:0051028	mRNA transport	1.39E-04	5/173	RANBP2|CASC3|AHCTF1|U2AF2|SRSF1	0	5
GO:0071826	ribonucleoprotein complex subunit organization	1.47E-04	6/288	LUC7L|SRSF1|GEMIN4|GEMIN5|DDX20|SRPK2	2	4
GO:0051272	positive regulation of cellular component movement	1.48E-04	8/577	RRAS2|HSPB1|VIL1|ACTN4|ILK|IRS1|AKT2|SPAG9	4	5
GO:0006260	DNA replication	1.96E-04	7/444	DKC1|NCOA6|MCM2|DNAJA3|TNKS1BP1|DNAJC2|NAP1L1	2	5
GO:0050792	regulation of viral process	2.08E-04	5/191	SNX3|GTF2F1|VAPB|TOP2A|SRPK2	1	4
GO:0006334	nucleosome assembly	2.15E-04	5/193	NAP1L1|MCM2|HJURP|H4C9|H4C1|H4C4|H4C6|H4C12|H4C11|H4C3|H4C8|H4C2|H4C5|H4C13|H4C14|H4-16|H4C15|HMGB2	2	3
GO:0007067	mitotic nuclear division	2.48E-04	6/321	ERCC6L|CLIP1|WAPL|DYNC1LI1|NUMA1|ZWILCH	2	4
GO:0000280	nuclear division	2.52E-04	7/467	ERCC6L|CLIP1|WAPL|DYNC1LI1|MKI67|NUMA1|ZWILCH	2	5
GO:0050657	nucleic acid transport	2.75E-04	5/207	RANBP2|CASC3|AHCTF1|U2AF2|SRSF1	0	5
GO:0050658	RNA transport	2.75E-04	5/207	RANBP2|CASC3|AHCTF1|U2AF2|SRSF1	0	5

Table S3. continued

GOID	Description	P-value	Hits	Gene List	Number of genes proteomics	Number of genes Phosphor-proteomics
GO:0051236	establishment of RNA localization	2.75E-04	5/207	RANBP2|CASC3|AHCTF1|U2AF2|SRSF1	0	5
GO:0034976	response to endoplasmic reticulum stress	2.75E-04	5/206	DNAJC10|VAPB|DNAJB11|ADD1|HSP90B1	3	2
GO:0007173	epidermal growth factor receptor signaling pathway	2.97E-04	5/211	AKT1S1|ITPR3|SH3KBP1|IRS1|VIL1	1	4
GO:0038127	ERBB signaling pathway	3.20E-04	5/215	AKT1S1|ITPR3|SH3KBP1|IRS1|VIL1	1	4
GO:0007163	establishment or maintenance of cell polarity	3.29E-04	5/217	MYO9B|ILK|CDC42BPB|MYH9|DLG5	1	4
GO:0010608	posttranscriptional regulation of gene expression	3.67E-04	8/676	DNAJA3|CNOT1|HSPB1|SRSF1|DNAJC1|ATP1B3|CASC3|AKT2	4	4
GO:0010563	negative regulation of phosphorus metabolic process	3.95E-04	8/686	AKT1S1|SFN|DNAJA3|GRB10|HSPB1|DNAJC10|PDE2A|ILK	5	3
GO:0045936	negative regulation of phosphate metabolic process	3.95E-04	8/686	AKT1S1|SFN|DNAJA3|GRB10|HSPB1|DNAJC10|PDE2A|ILK	5	3
GO:0031400	negative regulation of protein modification process	4.39E-04	8/698	AKT1S1|DNMT1|SFN|DNAJA3|HSPB1|DNAJC10|PSMD12|ILK	6	2
GO:0065004	protein-DNA complex assembly	4.56E-04	5/236	NAP1L1|MCM2|HJURP|H4C9|H4C1|H4C4|H4C6|H4C12|H4C11|H4C3|H4C8|H4C2|H4C5|H4C13|H4C14|H4-16|H4C15|HMGB2	2	3
GO:0051347	positive regulation of transferase activity	5.11E-04	9/910	SPAG9|DNAJB11|VAPB|HSP90B1|GHR|ILK|IRS1|ADD1|PSMD12	6	4
GO:2000147	positive regulation of cell motility	5.89E-04	7/553	HSPB1|VIL1|RRAS2|ILK|IRS1|AKT2|SPAG9	4	4

Table S3. continued

GOID	Description	P-value	Hits	Gene List	Number of genes proteomics	Number of genes Phosphor-proteomics
GO:0006417	regulation of translation	7.02E-04	6/405	CNOT1|HSPB1|SRSF1|DNAJC1|CASC3|AKT2	2	4
GO:0016197	endosomal transport	7.67E-04	5/267	AP1G1|VPS51|VPS25|SPAG9|VPS37A	4	2
GO:0043903	regulation of symbiosis, encompassing mutualism through parasitism	7.98E-04	5/270	SNX3|GTF2F1|VAPB|TOP2A|SRPK2	1	4
GO:0051348	negative regulation of transferase activity	8.38E-04	6/423	AKT1S1|SFN|DNAJA3|HSPB1|PSMD12|ILK	4	2
GO:0043161	proteasome-mediated ubiquitin-dependent protein catabolic process	8.38E-04	5/275	DNAJC10|RAD23B|HSP90B1|RFFL|PSMD12	3	2
GO:0040017	positive regulation of locomotion	8.38E-04	7/593	HSPB1|VIL1|RRAS2|ILK|IRS1|AKT2|SPAG9	4	4
GO:0007169	transmembrane receptor protein tyrosine kinase signaling pathway	8.77E-04	9/993	AKT1S1|GRB10|SH3KBP1|HSPB1|ITPR3|VIL1|GHR|IRS1|AKT2	3	6
GO:0060627	regulation of vesicle-mediated transport	9.37E-04	7/607	AP1G1|RAB27B|SNX3|ADD1|ACTN4|RAB21|AKT2	6	1
GO:0010498	proteasomal protein catabolic process	9.54E-04	5/285	DNAJC10|RAD23B|HSP90B1|RFFL|PSMD12	3	2
GO:0033674	positive regulation of kinase activity	9.81E-04	8/806	DNAJB11|VAPB|HSP90B1|GHR|ILK|IRS1|ADD1|SPAG9	5	4
GO:0042326	negative regulation of phosphorylation	1.00E-03	7/617	AKT1S1|SFN|DNAJA3|GRB10|HSPB1|DNAJC10|ILK	4	3
GO:0006469	negative regulation of protein kinase activity	1.02E-03	5/291	AKT1S1|ILK|DNAJA3|SFN|HSPB1	3	2

Table S3. continued

GOID	Description	P-value	Hits	Gene List	Number of genes	
					proteomics	Phosphor-proteomics
GO:0006366	transcription from RNA polymerase II promoter	1.05E-03	6/448	PHRF1|RBMX|GTF2F1|U2AF2|SRSF1|BTF3	1	5
GO:0060249	anatomical structure homeostasis	1.24E-03	6/465	DKC1|H4C9|H4C1|H4C4|H4C6|H4C12|H4C11|H4C3|H4C8|H4C2|H4C5|H4C13|H4C14|H4-16|H4C15|TNKS1BP1|HSPB1|LIG1|ADD1	1	5
GO:0033036	macromolecule localization	1.24E-03	8/842	DNAJA3|DLG5|WAPL|ACTN4|ATP1B3|CASC3|IRS1|AKT2	3	5
GO:0045216	cell-cell junction organization	1.28E-03	5/309	CTNND1|GNPAT|DLG5|GJA1|ACTN4	2	4
GO:0033673	negative regulation of kinase activity	1.56E-03	5/324	AKT1S1|ILK|DNAJA3|SFN|HSPB1	3	2
GO:0043900	regulation of multi-organism process	1.58E-03	6/490	AP1G1|GTF2F1|TOP2A|SNX3|VAPB|SRPK2	2	4
GO:0001701	in utero embryonic development	1.60E-03	7/678	GJA1|TBX3|SRSF1|MYH9|BTF3|ADD1|CHD8	2	5
GO:0030099	myeloid cell differentiation	1.65E-03	5/331	NCOA6|PDE2A|MYH9|ADD1|ACIN1	2	3
GO:0001933	negative regulation of protein phosphorylation	1.65E-03	6/497	AKT1S1|SFN|DNAJA3|HSPB1|DNAJC10|ILK	4	2
GO:0034660	ncRNA metabolic process	1.82E-03	7/697	NOC4L|DKC1|SRRT|GEMIN4|GEMIN5|DDX20|UTP18	2	5
GO:0030029	actin filament-based process	1.88E-03	7/703	MYO18A|SDCBP|VIL1|MYO9B|CDC42BPB|ADD1|MYH9	4	3
GO:0043009	chordate embryonic development	1.88E-03	7/703	GJA1|TBX3|SRSF1|MYH9|BTF3|ADD1|CHD8	2	5
GO:0009792	embryo development ending in birth or egg hatching	2.05E-03	7/715	GJA1|TBX3|SRSF1|MYH9|BTF3|ADD1|CHD8	2	5
GO:0034765	regulation of ion transmembrane transport	2.05E-03	5/352	AHNAK|ATP1B3|AKT2|GJA1|ACTN4	2	3

Table S3. continued

GOID	Description	P-value	Hits	Gene List	Number of genes	
					proteomics	Phosphor-proteomics
GO:0043086	negative regulation of catalytic activity	2.05E-03	8/927	AKT1S1|BCL2L12|SFN|DNAJA3|HSPB1|PSMD12|RFFL|ILK	4	4
GO:0030335	positive regulation of cell migration	2.38E-03	6/541	HSPB1|VIL1|RRAS2|ILK|IRS1|SPAG9	3	4
GO:0044262	cellular carbohydrate metabolic process	2.40E-03	5/367	PPP1CA|PGM2|LDHB|AKT2|IMPAD1	5	0
GO:0034762	regulation of transmembrane transport	2.40E-03	5/367	AHNAK|ATP1B3|AKT2|GJA1|ACTN4	2	3
GO:0071363	cellular response to growth factor stimulus	2.43E-03	8/959	AKT1S1|NCOR2|PPP1CA|HSPB1|ITPR3|VIL1|PDE2A|IRS1	3	5
GO:0051179	localization	2.66E-03	8/974	DNAJA3|DLG5|WAPL|ACTN4|ATP1B3|CASC3|IRS1|AKT2	3	5
GO:0045860	positive regulation of protein kinase activity	2.86E-03	7/768	DNAJB11|VAPB|HSP90B1|GHR|ILK|ADD1|SPAG9	5	3
GO:0034470	ncRNA processing	2.96E-03	5/389	NOC4L|DKC1|SRRT|UTP18|GEMIN4	2	3
GO:0019941	modification-dependent protein catabolic process	2.96E-03	7/775	ZMPSTE24|VPS37A|DNAJC10|RAD23B|PSMD12|RFFL|HSP90B1	4	3
GO:0043632	modification-dependent macromolecule catabolic process	3.02E-03	7/779	ZMPSTE24|VPS37A|DNAJC10|RAD23B|PSMD12|RFFL|HSP90B1	4	3
GO:0007411	axon guidance	3.21E-03	6/583	PGRMC1|KIF4A|SDCBP|ENAH|HSP90AA1|MYH9	2	4
GO:0097485	neuron projection guidance	3.24E-03	6/585	PGRMC1|KIF4A|SDCBP|ENAH|HSP90AA1|MYH9	2	4
GO:0051259	protein oligomerization	3.40E-03	7/799	AHNAK|GJA1|RBMX|ITPR3|ST13|SPAG9|ILK	2	6
GO:0009790	embryo development	3.61E-03	7/809	GJA1|TBX3|SRSF1|MYH9|BTF3|ADD1|CHD8	2	5
GO:0008104	protein localization	3.94E-03	7/823	DNAJA3|DLG5|WAPL|ACTN4|ATP1B3|IRS1|AKT2	3	4
GO:0030855	epithelial cell differentiation	4.08E-03	6/617	KRT10|VDAC1|DLG5|VIL1|NUMA1|AKT2	4	2

Table S3. continued

GOID	Description	P-value	Hits	Gene List	Number of genes	
					proteomics	Phosphor-proteomics
GO:0030163	protein catabolic process	4.10E-03	5/426	DNAJC10|RAD23B|HSP90B1|RFFL|PSMD12	3	2
GO:0007015	actin filament organization	4.11E-03	5/427	ADD1|ENAH|HSP90B1|ACTN4|VIL1	3	2
GO:0030111	regulation of Wnt signaling pathway	4.77E-03	5/443	ILK|GRB10|MACF1|CTNND1|CHD8	2	4
GO:0051603	proteolysis involved in cellular protein catabolic process	4.94E-03	7/864	ZMPSTE24|VPS37A|DNAJC10|RAD23B|PSMD12|RFFL|HSP90B1	4	3
GO:0030162	regulation of proteolysis	5.13E-03	6/652	BCL2L12|SFN|DNAJA3|DNAJC1|RAD23B|RFFL	3	3
GO:0070727	cellular macromolecule localization	5.48E-03	6/662	DNAJA3|WAPL|ATP1B3|CASC3|IRS1|AKT2	3	3
GO:2001233	regulation of apoptotic signaling pathway	5.76E-03	6/671	SFN|SFPQ|PPP1CA|HSPB1|HMGB2|RFFL	4	2
GO:0010948	negative regulation of cell cycle process	5.76E-03	5/467	TOP2A|WAPL|PSMD12|ZWILCH|TOP2B	2	3
GO:0044087	regulation of cellular component biogenesis	5.93E-03	7/900	GJA1|CNOT1|CLIP1|MACF1|HJURP|ADD1|VIL1	3	4
GO:0071417	cellular response to organonitrogen compound	7.05E-03	7/931	DNMT1|GRB10|PDE2A|HSP90B1|GHR|IRS1|AKT2	4	3
GO:0071705	nitrogen compound transport	7.43E-03	6/711	GJA1|AHCTF1|RANBP2|U2AF2|SRSF1|CASC3	0	6
GO:0051098	regulation of binding	7.46E-03	5/501	SPAG9|WAPL|HJURP|ADD1|HMGB2	3	3
GO:0070613	regulation of protein processing	8.84E-03	6/740	BCL2L12|SFN|DNAJA3|DNAJC1|RAD23B|RFFL	3	3
GO:0010564	regulation of cell cycle process	9.12E-03	7/983	TOP2A|TOP2B|WAPL|PSMD12|AKT2|ZWILCH|DYNC1LI1	4	3

Table S3. continued

GOID	Description	P-value	Hits	Gene List	Number of genes	
					proteomics	Phosphor-proteomics
GO:0051052	regulation of DNA metabolic process	9.64E-03	5/536	MSH6|WAPL|DNAJC2|TOP2A|GJA1	0	5
GO:0006511	ubiquitin-dependent protein catabolic process	9.68E-03	6/758	VPS37A|DNAJC10|RAD23B|HSP90B1|RFFL|PSMD12	3	3
GO:0002768	immune response-regulating cell surface receptor signaling pathway	1.09E-02	5/554	AKT1S1|ITPR3|ENAH|HSP90AA1|IRS1	1	4
GO:0050817	coagulation	1.12E-02	5/560	ATP1B3|KIF4A|ITPR3|ACTN4|PDE2A	2	3
GO:0007596	blood coagulation	1.12E-02	5/560	ATP1B3|KIF4A|ITPR3|ACTN4|PDE2A	2	3
GO:0002764	immune response-regulating signaling pathway	1.13E-02	6/788	AKT1S1|ITPR3|ENAH|HSP90B1|IRS1|HSP90AA1	1	5
GO:0048858	cell projection morphogenesis	1.13E-02	5/563	MYO9B|TOP2B|MAP1S|GJA1|ILK	1	4
GO:0016337	single organismal cell-cell adhesion	1.13E-02	5/565	ILK|CTNND1|HSPB1|DLG5|MYH9	2	4
GO:0051641	cellular localization	1.13E-02	6/792	DNAJA3|WAPL|ATP1B3|CASC3|IRS1|AKT2	3	3
GO:0007599	hemostasis	1.15E-02	5/569	ATP1B3|KIF4A|ITPR3|ACTN4|PDE2A	2	3
GO:0051223	regulation of protein transport	1.17E-02	6/801	SFN|MYO18A|DNAJC1|SNX3|PDE2A|AKT2	5	1
GO:0050878	regulation of body fluid levels	1.39E-02	6/834	SFN|KIF4A|ITPR3|ACTN4|PDE2A|ATP1B3	3	3
GO:1900542	regulation of purine nucleotide metabolic process	1.39E-02	6/834	AKAP12|DNAJC10|DNAJC1|PDE2A|ATP1B3|AKT2	4	2
GO:0006140	regulation of nucleotide metabolic process	1.41E-02	6/837	AKAP12|DNAJC10|DNAJC1|PDE2A|ATP1B3|AKT2	4	2

Table S3. continued

GOID	Description	P-value	Hits	Gene List	Number of genes proteomics	Number of genes Phosphor-proteomics
GO:0032990	cell part morphogenesis	1.43E-02	5/606	MYO9B|TOP2B|MAP1S|GJA1|ILK	1	4
GO:0046486	glycerolipid metabolic process	1.49E-02	5/613	PPP1CA|CDS2|GNPAT|IMPAD1|CDIPT	4	1
GO:0098602	single organism cell adhesion	1.51E-02	5/616	ILK|CTNND1|HSPB1|DLG5|MYH9	2	4
GO:0030036	actin cytoskeleton organization	1.61E-02	5/627	SDCBP|CDC42BPB|MYH9|ADD1|MYO18A	3	2
GO:0070201	regulation of establishment of protein localization	1.73E-02	6/883	SFN|MYO18A|DNAJC1|SNX3|PDE2A|AKT2	5	1
GO:0051051	negative regulation of transport	1.75E-02	5/643	GRB10|SNX3|IRS1|AKT2|PDE2A	3	2
GO:0006644	phospholipid metabolic process	1.76E-02	5/645	CDIPT|SGPP1|GNPAT|IMPAD1|CDS2	3	2
GO:0000226	microtubule cytoskeleton organization	1.82E-02	5/652	NUMA1|CLIP1|SON|MAP1S|MYH9	0	5
GO:0034613	cellular protein localization	1.82E-02	5/651	ATP1B3|WAPL|IRS1|AKT2|DNAJA3	3	2
GO:0080135	regulation of cellular response to stress	2.00E-02	5/670	SDCBP|SPAG9|HMGA1|HSPB1|SFPQ	3	3
GO:0007346	regulation of mitotic cell cycle	2.23E-02	5/690	TOP2A|TOP2B|DYNC1LI1|ZWILCH|PSMD12	3	2
GO:0008610	lipid biosynthetic process	2.39E-02	6/960	CDS2|NSDHL|GNPAT|VAPB|CDIPT|SGPP1	3	3
GO:0032386	regulation of intracellular transport	2.56E-02	5/718	SNX3|RAB21|SFN|AKT2|PDE2A	5	0
GO:0032446	protein modification by small protein conjugation	2.57E-02	5/720	UBR4|RANBP2|PSMD12|RFFL|HUWE1	2	3

Table S3. continued

GOID	Description	P-value	Hits	Gene List	Number of genes proteomics	Number of genes Phosphor-proteomics
GO:0045087	innate immune response	2.57E-02	6/981	AKT1S1|ITPR3|HSP90B1|IRS1|SRPK2|HSP90AA1	0	6
GO:0010638	positive regulation of organelle organization	2.61E-02	5/726	CLIP1|DNMT1|SFN|AKT2|VIL1	4	1
GO:0002009	morphogenesis of an epithelium	2.61E-02	5/725	TBX3|PPP1CA|DLG5|GJA1|ILK	2	3
GO:0022604	regulation of cell morphogenesis	3.18E-02	5/767	ILK|LARP4|SH3KBP1|MYH9|VIL1	2	3
GO:0043269	regulation of ion transport	3.24E-02	5/772	AHNAK|ATP1B3|AKT2|GJA1|ACTN4	2	3
GO:0043065	positive regulation of apoptotic process	3.61E-02	5/797	DDX20|ACIN1|TOP2A|SRPK2|DNAJA3	1	4
GO:0071345	cellular response to cytokine stimulus	3.69E-02	5/803	MCM2|RANBP2|PLP2|PDE2A|RBMX	3	2
GO:0043068	positive regulation of programmed cell death	3.71E-02	5/805	DDX20|ACIN1|TOP2A|SRPK2|DNAJA3	1	4
GO:0043434	response to peptide hormone	3.75E-02	5/809	GRB10|AKT2|IRS1|GJA1|GHR	2	3
GO:0051707	response to other organism	4.14E-02	5/833	GTF2F1|HMGA1|HSPB1|HNRNPUL1|VIL1	1	4
GO:0070647	protein modification by small protein conjugation or removal	4.37E-02	5/847	UBR4|RANBP2|PSMD12|RFFL|HUWE1	2	3
GO:0010942	positive regulation of cell death	4.51E-02	5/856	DDX20|ACIN1|TOP2A|SRPK2|DNAJA3	1	4
GO:1901652	response to peptide	4.87E-02	5/876	GRB10|AKT2|IRS1|GJA1|GHR	2	3

Table S4. Signaling pathway enrichment analysis of siZNF322A phosphoproteomics in A549 lung cancer cells by REACTOME.

Pathway name	Entities found	Entities total	Entities pValue	Entities FDR	Mapped entities
mRNA Splicing - Major Pathway	7	135	6.69E-04	0.19313	SRSF1;SRRT;U2AF2;CASC3;GTF2F1;HNRNPUL1;RBMX
mRNA Splicing	7	142	8.97E-04	0.19313	SRSF1;SRRT;U2AF2;CASC3;GTF2F1;HNRNPUL1;RBMX
Processing of Capped Intron-Containing Pre-mRNA	8	195	1.25E-03	0.19313	SRSF1;SRRT;U2AF2;RANBP2;CASC3;GTF2F1;HNRNPUL1;RBMX
IRS activation	2	5	1.38E-03	0.19313	GRB10;IRS1
Cellular response to heat stress	5	93	3.42E-03	0.38355	AKT1S1;HSP90AA1;DNAJC2;RANBP2;ST13
Signal attenuation	2	10	5.33E-03	0.40796	GRB10;IRS1
Transport of Mature mRNA derived from an Intron-Containing Transcript	4	72	7.80E-03	0.40796	SRSF1;RANBP2;U2AF2;CASC3
Resolution of Sister Chromatid Cohesion	5	120	9.78E-03	0.40796	RANBP2;CLIP1;WAPL;AHCTF1;ERCC6L
Mismatch repair (MMR) directed by MSH2:MSH6 (MutSalpha)	2	14	1.02E-02	0.40796	MSH6;LIG1
Transport of Mature Transcript to Cytoplasm	4	78	1.02E-02	0.40796	SRSF1;RANBP2;U2AF2;CASC3
Cell Cycle	13	591	1.08E-02	0.40796	HSP90AA1;DKC1;RANBP2;CLIP1;TOP2A;HJURP;LIG1;WAPL;TMPO;AHCTF1;NUMA1;ERCC6L;H4C1
Mismatch Repair	2	15	1.16E-02	0.40796	MSH6;LIG1
Mitotic Prometaphase	5	128	1.26E-02	0.40796	RANBP2;CLIP1;WAPL;AHCTF1;ERCC6L
Chromosome Maintenance	4	86	1.42E-02	0.40796	DKC1;HJURP;LIG1;H4C1
SUMOylation of DNA replication proteins	3	48	1.53E-02	0.40796	TOP2B;RANBP2;TOP2A
Cell Cycle, Mitotic	11	487	1.57E-02	0.40796	HSP90AA1;RANBP2;CLIP1;TOP2A;LIG1;WAPL;TMPO;AHCTF1;NUMA1;ERCC6L;H4C1
mRNA 3'-end processing	3	51	1.79E-02	0.40796	SRSF1;U2AF2;CASC3
Mitotic Anaphase	6	195	1.90E-02	0.40796	RANBP2;CLIP1;WAPL;TMPO;AHCTF1;ERCC6L
Mitotic Metaphase and Anaphase	6	196	1.94E-02	0.40796	RANBP2;CLIP1;WAPL;TMPO;AHCTF1;ERCC6L
snRNP Assembly	2	22	2.37E-02	0.40796	DDX20;GEMIN5

Table S4. continued

Pathway name	Entities found	Entities total	Entities pValue	Entities FDR	Mapped entities
Metabolism of non-coding RNA	2	22	2.37E-02	0.40796	DDX20;GEMIN5
Telomere Maintenance	3	59	2.61E-02	0.40796	DKC1;LIG1;H4C1
Cleavage of Growing Transcript in the Termination Region	3	60	2.73E-02	0.40796	SRSF1;U2AF2;CASC3
RNA Polymerase II Transcription Termination	3	60	2.73E-02	0.40796	SRSF1;U2AF2;CASC3
HSF1-dependent transactivation	2	24	2.79E-02	0.40796	AKT1S1;HSP90AA1
Regulation of gap junction activity	1	3	3.17E-02	0.40796	GJA1
c-src mediated regulation of Cx43 function and closure of gap junctions	1	3	3.17E-02	0.40796	GJA1
Transport of connexins along the secretory pathway	1	3	3.17E-02	0.40796	GJA1
Oligomerization of connexins into connexons	1	3	3.17E-02	0.40796	GJA1
VEGFR2 mediated vascular permeability	2	27	3.45E-02	0.40796	HSP90AA1;CTNND1
Extension of Telomeres	2	28	3.69E-02	0.40796	DKC1;LIG1
YAP1- and WWTR1 (TAZ)-stimulated gene expression	2	29	3.93E-02	0.40796	NCOA6;YAP1
M Phase	7	297	4.14E-02	0.40796	CLIP1;RANBP2;WAPL;TMPO;AHCTF1;ERCC6L;H4C1
Regulation of HSF1-mediated heat shock response	3	73	4.45E-02	0.40796	DNAJC2;RANBP2;ST13
RNA Polymerase II Transcription	4	124	4.52E-02	0.40796	SRSF1;U2AF2;CASC3;GTF2F1
Separation of Sister Chromatids	5	183	4.79E-02	0.40796	RANBP2;CLIP1;WAPL;AHCTF1;ERCC6L
